# Quantitative Outcomes of a One Health approach to Study Global Health Challenges

**DOI:** 10.1007/s10393-017-1310-5

**Published:** 2018-01-12

**Authors:** Laura C. Falzon, Isabel Lechner, Ilias Chantziaras, Lucie Collineau, Aurélie Courcoul, Maria-Eleni Filippitzi, Riikka Laukkanen-Ninios, Carole Peroz, Jorge Pinto Ferreira, Merel Postma, Pia G. Prestmo, Clare J. Phythian, Eleonora Sarno, Gerty Vanantwerpen, Timothée Vergne, Douglas J. C. Grindlay, Marnie L. Brennan

**Affiliations:** 1 Veterinary Public Health Institute, University of Bern, Schwarzenburgstrasse 155, 3097 Liebefeld, Switzerland; 2 Institute of Infection and Global Health, University of Liverpool, 8 West Derby Street, Liverpool, L69 7BE UK; 3 Veterinary Epidemiology Unit, Department of Reproduction, Obstetrics and Herd Health, Faculty of Veterinary Medicine, Ghent University, Salisburylaan 133, 9820 Merelbeke, Belgium; 4 SAFOSO, Waldeggstrasse 1, 3097 Liebefeld, Switzerland; 5 Anses, Laboratory of Animal Health, Epidemiology Unit, University Paris Est, 23, Avenue du Général de Gaulle, 94706 Maisons-Alfort Cedex, France; 6 Department of Food Hygiene and Environmental Health, Faculty of Veterinary Medicine, University of Helsinki, P.O. Box 66, 00014 Helsinki, Finland; 7 BIOEPAR, INRA, Oniris, 44307 Nantes, France; 8 School of Veterinary Sciences, University of Bristol, Langford House, Langford, Bristol, BS40 5DU UK; 9 Section for Small Ruminant Research, Faculty of Veterinary Medicine, Institute for Production Animal Clinical Science, Norwegian University of Life Sciences, 4325 Sandnes, Norway; 10 Institute for Food Safety and Hygiene, University of Zurich, Winterthurerstrasse 272, 8057 Zürich, Switzerland; 11 Department of Veterinary Public Health and Food Safety, Faculty of Veterinary Medicine, Ghent University, Merelbeke, Belgium; 12 Veterinary Epidemiology Economics and Public Health group, Royal Veterinary College, London, UK; 13 MIVEGEC Group, Institut de Recherche pour le développement, Montpellier, France; 14 UMR ENVT-INRA IHAP, University of Toulouse, Toulouse, France; 15 Centre of Evidence-based Dermatology, The University of Nottingham, King’s Meadow Campus, Nottingham, NG7 2NR UK; 16 Centre for Evidence-based Veterinary Medicine, School of Veterinary Medicine and Science, The University of Nottingham, Sutton Bonington Campus, Loughborough, LE12 5RD UK

**Keywords:** One Medicine, Transdisciplinarity, Endemic and emerging infectious diseases, Zoonoses, Non-communicable diseases, Systematic evidence, Scoping review

## Abstract

Having gained momentum in the last decade, the One Health initiative promotes a holistic approach to address complex global health issues. Before recommending its adoption to stakeholders, however, it is paramount to first compile quantitative evidence of the benefit of such an approach. The aim of this scoping review was to identify and summarize primary research that describes monetary and non-monetary outcomes following adoption of a One Health approach. An extensive literature search yielded a total of 42,167 references, of which 85 were included in the final analysis. The top two biotic health issues addressed in these studies were rabies and malaria; the top abiotic health issue was air pollution. Most studies described collaborations between human and animal (*n* = 42), or human and environmental disciplines (*n* = 41); commonly reported interventions included vector control and animal vaccination. Monetary outcomes were commonly expressed as cost–benefit or cost–utility ratios; non-monetary outcomes were described using disease frequency or disease burden measurements. The majority of the studies reported positive or partially positive outcomes. This paper illustrates the variety of health challenges that can be addressed using a One Health approach, and provides tangible quantitative measures that can be used to evaluate future implementations of the One Health approach.

**Electronic supplementary material:**

The online version of this article (10.1007/s10393-017-1310-5) contains supplementary material, which is available to authorized users.

## Introduction

The One Health (OH) approach is based on the notion that human, animal, and environmental health are intimately connected and mutually dependent (Rabinowitz et al. 2008; Dixon et al. 2014). Consequently, advocates of this movement describe the need for a holistic and transdisciplinary approach when tackling complex global health issues with high societal values (American Veterinary Medical Association 2008; Greter et al. 2014).

Despite being considered by some as a novel approach, the concept of OH dates back many centuries (Oura 2014; Woods and Bresalier 2014). Several key figures have played an important role in the promotion of this approach, through recognition of the similarities between human and veterinary medical science, the study of zoonoses and vaccine discovery, and the coining of the terms “One Medicine,” “One Health,” and “Ecohealth” (Day 2010; Zinsstag et al. 2011; Murray et al. 2014; Roberts 2014; Woods and Bresalier 2014). More recent key events in the OH movement include the publication of the Manhattan Principles recognizing the importance of a holistic approach when tackling both epidemic and epizootic diseases (World Conservation Society 2004) and the signing of the Tripartite Concept Note which puts onus on promoting prevention and control of disease at the human–animal–ecosystem interface (The FAO-OIE-WHO Collaboration 2010).

While the benefits of such a holistic and integrative movement may seem intuitive, the OH approach has come under scrutiny for its accountability, particularly since further investment in such collaborative projects will require a change in the way funds are allocated (Cleaveland et al. 2014; Gibbs 2014). Currently, most funds are administered within sectors. Yet, the collaborative approaches and applications encouraged by the OH movement often require a substantial initial investment which may go well beyond the possibilities of independent sectors or institutions. Therefore, to allow for more researchers to embrace this approach, there is a need to create interministerial platforms which allow for more integrated surveillance and disease control programs involving the animal, human, and environmental sectors, or novel funding mechanisms which will provide and accommodate for this transdisciplinary approach (Häsler et al. 2012; Gibbs 2014). For example, to prevent human disease and mitigate agricultural damages, a solution may lie primarily with more effective animal vaccination programs, requiring commitment and cohesion across disciplines. However, for this paradigm shift to occur, funding agencies and policy-makers must be provided with more evidence on the added value and cost-effectiveness of such cross-sectorial approaches (Hodgson and Darling 2011; Häsler et al. 2012; The World Bank 2012; Boden et al. 2014).

Therefore, the aim of this scoping review (SR) was (1) to systematically identify those studies that describe a quantitative outcome when using a OH approach and (2) to review and qualitatively summarize the health issues addressed, the type of OH approaches used, and the nature and value of the quantitative outcomes described. The purpose of this study is to create an evidence base of the types of OH applications, and consequent monetary and non-monetary outcomes accrued.

## Methods

### Research Question, Definitions, and Protocol

This SR was conducted to identify and summarize studies which describe a quantitative outcome when using a OH approach to address complex global health challenges. The study was performed as a joint project among residents of the European College of Veterinary Public Health. The population of interest within the studies was defined as the human and animal population worldwide. The intervention of interest was the “OH approach,” defined as “the collaborative efforts of multiple disciplines working locally, nationally and globally to attain optimal health for people, animals and our environment” (American Veterinary Medical Association 2008). The outcome of interest was a “quantitative outcome,” measured either in monetary or non-monetary terms (Rusthon 2009; Rushton et al. 2012; Minutes of the expert workshop 2013).

An *a priori* protocol was developed to define eligibility criteria and procedure after consultation with experts in OH and veterinary economics. Additional references were used to help structure the SR (Higgins and Green 2008; Centre for reviews and dissemination 2009), which is reported according to PRISMA guidelines (Moher et al. 2009). Screening tools (S1 and S2) were pretested before implementation to ensure clarity of questions.

### Literature Search Strategy

**Fig. 1:**
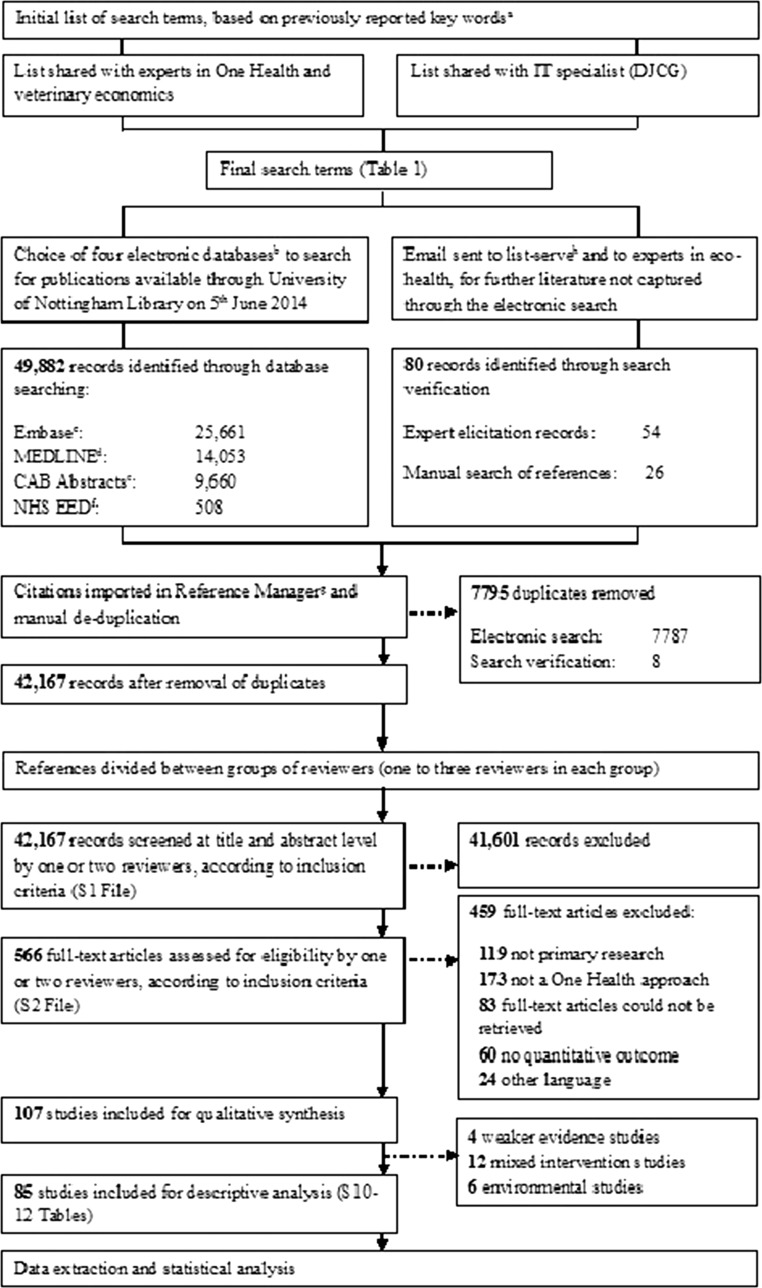
Flow of methodological activities and information through the different phases of a scoping review on the quantitative outcome of a One Health approach to address complex global health challenges, as described by the PRISMA guidelines (Moher et al. 2009). ^a^Keywords reported in Rushton 2009; Häsler et al. 2012; Minutes of the Expert Workshop 2013. ^b^Based on a recommendation that three to five databases are considered sufficient (Young et al. 2014). ^c^Texts available between 1980 and 2014. ^d^Texts available between 1946 and 2014. ^e^Texts available between 1910 and 2014. ^f^Part of the National Institute for Health Research Centre for Reviews and Dissemination, UK. ^g^Refworks^©^ (ProQuest, LLC, Cambridge Information Group; Betheseda, MD, USA). ^h^Community of Practice in Ecosystem Approaches to Health—Canada.

The outline of the methodological activities undertaken is presented in Fig. 1. The search terms presented in Table 1 were used to systematically search four electronic databases: MEDLINE, CAB Abstracts, Embase, and the National Health Service Economic Evaluation Database (NHS EED; UK). The final search was performed on June 5, 2014, and the search strategies used for each database are presented in S3–S6. In addition to the electronic search, a search verification was performed through expert elicitation to help with the identification of relevant studies within the gray literature, and by manually searching references in recent reviews on the topic (Zinsstag et al. 2007; Zinsstag et al. 2011; Häsler et al. 2012; Min et al. 2013).

**Table 1:** A List of the Search Terms Used in Four Electronic Databases (MEDLINE, Embase, NHS EED, and CAB Abstracts) to Identify References that Describe a Quantitative Outcome when Using a One Health Approach to Address Complex Global Health Challenges.

((animal AND human) OR (animals and human) OR (animal AND humans) OR (animals AND humans) OR (human AND environment) OR (humans AND environment) OR (animal AND environment) OR (animals AND environment) OR “animal to human” OR “human to animal” OR “social-ecological” OR “socio-ecological” OR “One Health” OR “Ecohealth” OR “One World” OR “One Medicine” OR (ecosystem AND health) OR (holistic AND health) OR (veterinary AND human medicine) OR interdisciplinary OR multidisciplinary OR transdisciplinary OR “cross sector” OR “inter sector” OR “trans sector” OR zoonos* OR zoonotic OR “veterinary public health” OR “VPH” OR “farm to fork” OR “stable to table” OR “value chain”)
AND
(DALY* OR HALY* OR QALY* OR “disability adjusted life year” OR “disability adjusted life years” OR “health adjusted life year” OR “health adjusted life years” OR “quality adjusted life year” OR “quality adjusted life years” OR “expected quality adjusted life year” OR “expected quality adjusted life years” OR “opportunity cost” OR “opportunity costs” OR “cost benefit” OR “cost benefits” OR “cost analys*” OR “cost assessment” OR “cost effectiveness” OR “cost utility” OR “cost utilities” OR profit* OR “cost allocation” OR “cost benefit analys*” OR “cost control” OR “cost controls” OR “cost saving” OR “cost savings” OR “costs savings” OR “cost of illness” OR “costs of illness” OR “cost of disease” OR “costs of disease” OR “cost of intervention” OR “costs of intervention” OR “cost sharing” OR “costs sharing” OR “health care cost” OR “health care costs” OR “health care expenditure” OR “health care expenditures” OR “value of life” OR “societal benefit*” OR “economic evaluation” OR “economic analys*” OR “economic assessment” OR “health economics” OR “resource allocation” OR “cost avoidance” OR “costs avoidance” OR “loss avoidance” OR “losses avoidance”)

### Study Inclusion Criteria and Screening

The predetermined criteria for a publication to be eligible for inclusion are given in S1, while the screening strategy followed is shown in Fig. 1. A publication was considered eligible for inclusion if it reported primary research on a quantitative outcome when using a OH approach, even if not explicitly defined as such, to address complex global health challenges, and was published after 1910. This date was selected based on the setup of the databases, whereby the earliest publication date available was 1910. Primary research was defined as a study where the author(s) collected and/or analyzed data, and included case reports and case series, qualitative studies, observational studies, and experimental studies. Mathematical models and economic studies were included if they were based on field data collected in the same study or elsewhere. References were included if they were in English, German, Italian, Spanish, French, Portuguese, Greek, Dutch, Finnish, Russian, Norwegian, or Swedish; references in other languages were excluded. If no abstract was available, and the title was not sufficiently clear, the publication was included for full-text screening. Discrepancies regarding a publication’s eligibility were first resolved among the smaller group of reviewers and, when necessary, through an online discussion with all reviewers involved in this study.

### Qualitative Data Extraction and Analysis

Data extracted from the included publications are shown in S7-S12; these included: (1) bibliographic information and study design characteristics, (2) how the reference was identified, (3) the health issue addressed, (4) the intersectoral approach used (i.e., human–animal vs. human–environment vs. animal–environment vs. human–animal–environment), (5) the quantitative outcome described, and (6) a quality assessment based on the clarity of the methods. All extracted data were checked for consistency by two of the authors (LCF and MLB), and any disagreements were resolved through discussion between all reviewers.

To allow for further exploration and description of the studies, the following parameters were extracted: (1) continent where the study was performed; (2) whether the country was considered developed or developing, and its income status; (3) whether the disease agent was abiotic or biotic and, in case of the latter, whether it was a bacterium, virus, protozoa, helminth, or insect; (4) whether the health issue was considered a neglected tropical disease (NTD) or not; and (5) the type of transmission. The definitions of these parameters are based on references provided in S13. Descriptive statistics of the study characteristics (e.g., health issue described, type of intervention, and outcome) were performed using Stata (version 13, StataCorp LP, College Station, TX, USA). Due to the heterogeneity of the studies and topics involved, quantitative meta-analyses were not undertaken.

This review was approved by the Ethical Review Committee at the University of Nottingham, UK (Ethics Approval Number: 1328141209).

## Results

Figure 1 shows the flow of references through the screening process. Of the 107 studies that were included for qualitative synthesis, 4 were excluded because they showed elements of a OH approach, but multiple steps described the link between the OH approach and quantitative outcome, with certain overarching assumptions not explicitly discussed (S7). Twelve studies were excluded as “Mixed Interventions” because, while they described both interdisciplinary and disciplinary interventions, it was not possible to determine the quantitative outcome specifically due to the OH approach (S8). Another six studies that described a OH approach to address environmental health issues were classified separately (S9).

**Fig. 2:**
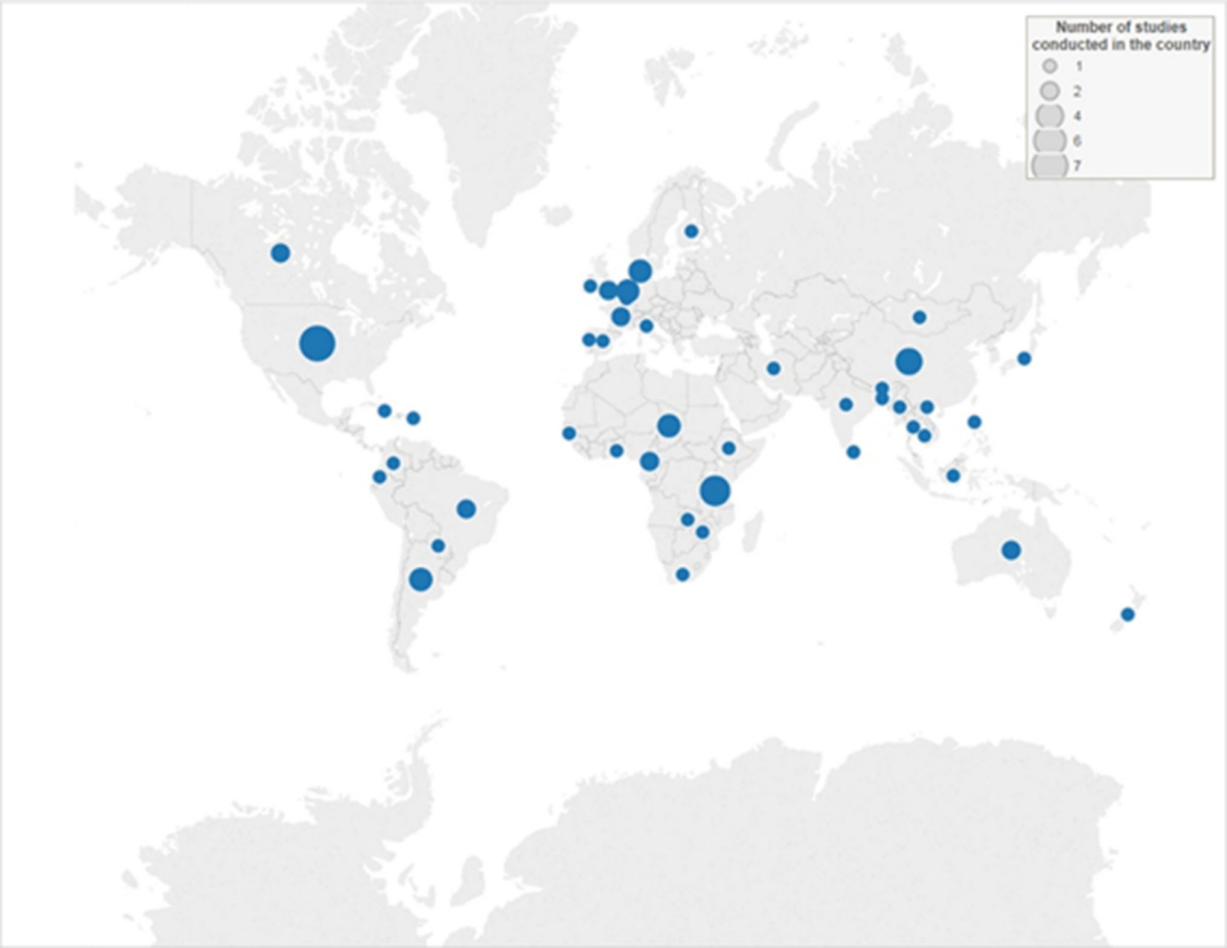
A world map indicating the number of studies conducted in different countries and included in a scoping review on the quantitative outcome of a One Health approach to address complex global health challenges.

The remaining 85 studies fully met our aim and eligibility criteria (S10–S12); of these, 72 were identified through the electronic search, while 13 were identified through search verification. The studies were performed in all five continents, primarily in Europe (*n* = 23), Asia (*n* = 20), and Africa (*n* = 16). A total of 56 different countries or regions were represented (Fig. 2), most commonly the USA (*n* = 7), China (*n* = 4), and Tanzania (*n* = 4). Thirty-six studies were performed in developed countries, while another 44 were performed in developing countries; the remaining 5 studies either did not specify the country, or were performed in countries (Cambodia and Puerto Rico) that did not appear within the reference document used for the classification of developing/developed status (United Nations 2014; see S13). Similarly, 37, 25, and 14 of these studies were performed in high-, middle-, and low-income countries, respectively.

The publication date of the included studies ranged between 1984 and 2014; the majority (*n* = 70) were published after 2000, of which 33 between 2010 and 2014. The majority of the included references described modeling studies such as economic analyses (*n* = 42), mathematical modeling (*n* = 12), and risk assessments (*n* = 4).

### Health Issues Addressed

**Fig. 3:**
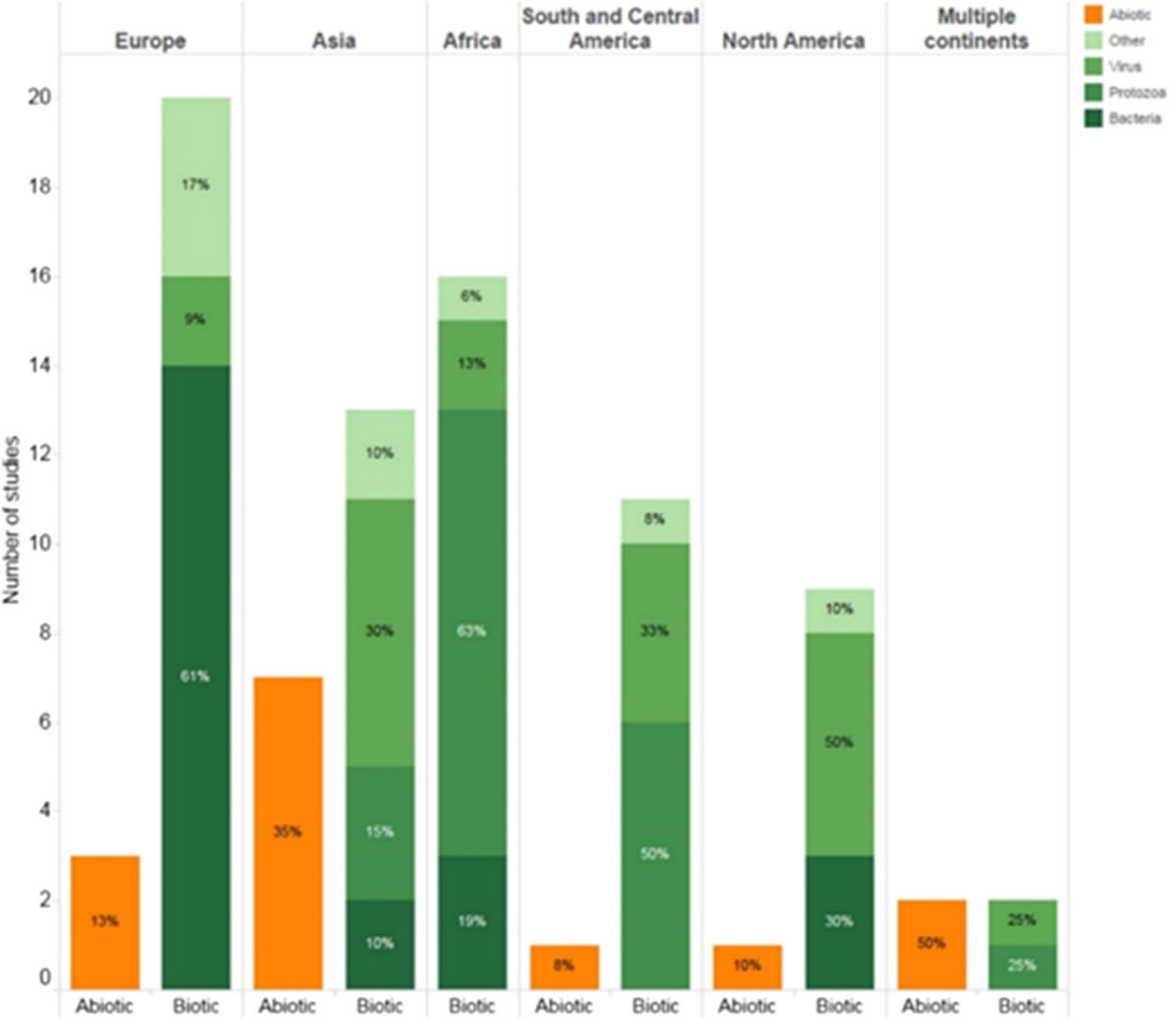
Abiotic and biotic health issues described, per continent, in a scoping review on the quantitative outcome of a One Health approach to address complex global health challenges.

**Fig. 4:**
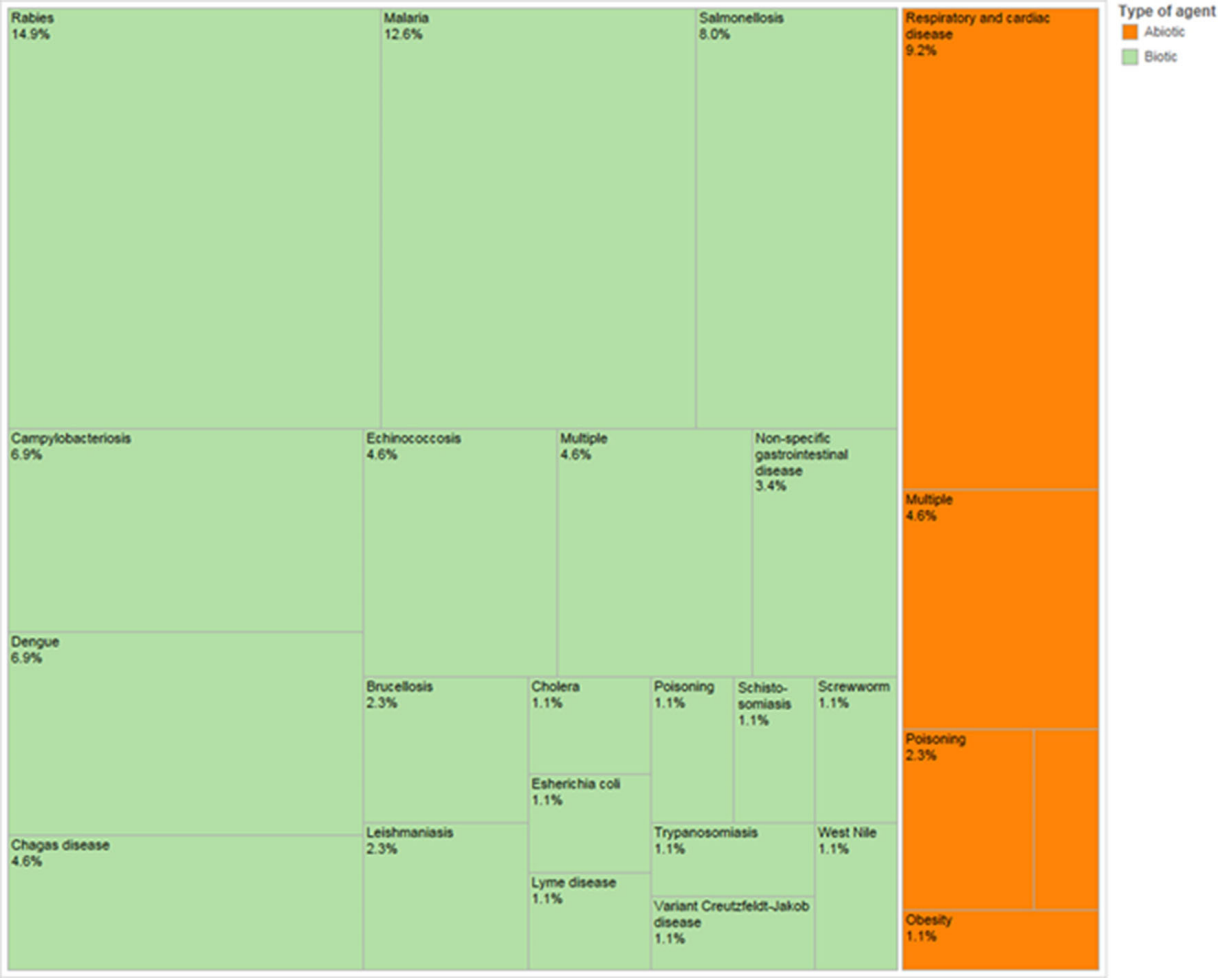
Abiotic and biotic health issues described in a scoping review on the quantitative outcome of a One Health approach to study complex health challenges.

The health issues addressed in the 85 studies were classified as biotic (*n* = 69), abiotic (*n* = 14), or both (*n* = 2; Figs. 3 and 4).

Among those studies that included a biotic issue, the top five diseases described were rabies (*n* = 13), malaria (*n* = 11), salmonellosis (*n* = 7), campylobacteriosis (*n* = 6), and dengue (*n* = 6). Almost half (*n* = 32) dealt with a NTD such as rabies, dengue, echinococcosis, and Chagas disease. Most of the bacterial studies were performed in Europe (*n* = 14), while most protozoal studies were performed in Africa (*n* = 10).

Air pollution was the most common abiotic health issue addressed (*n* = 5); other issues included pesticides, micro-pollutants in water, and exposure to heavy metals in water or soil. Most of the 14 studies investigating abiotic health issues were conducted after the year 2000 and were performed in Asia (*n* = 7) and Europe (*n* = 3).

### One Health Approach

The majority of these 85 studies either described a collaboration between human and animal (*n* = 42), or between human and environmental (*n* = 41) disciplines. Of all interventions, environmental interventions were the most commonly described, and these targeted vector control (*n* = 26), pollution (*n* = 8), sanitation and water (*n* = 8), or modified environmental spaces to encourage physical activity (*n* = 1). More specifically, vector control was achieved primarily through the use of insecticide-treated bed nets, control of breeding sites, and habitat restoration. Pollution and sanitation were largely controlled through policies and structural changes. Other interventions described included vaccination of domestic animals or wildlife (either singly or in combination with other interventions; *n* = 20), best management practices targeting primary production (*n* = 12), treatment (*n* = 6), integrated surveillance (*n* = 2), and combined human and animal physical activity (*n* = 2).

### Quantitative Outcomes

Of the studies included, some described both monetary and non-monetary outcomes (*n* = 31), while others described only monetary (*n* = 33) or non-monetary (*n* = 21) outcomes (S10-S12).

Most monetary outcomes were described as cost–benefit ratios (*n* = 26), cost–utility ratios (*n* = 18), or cost savings (*n* = 15). The majority of the studies had positive (*n* = 40) or partially positive (*n* = 18) monetary outcomes expressed as positive benefit–cost ratios and net present values, increased cost–utility ratios, or marked cost savings. Only four of the studies had a negative monetary outcome, expressed as negative benefit–to–cost ratios or imbalanced costs.

Among the non-monetary outcomes, measures of disease frequency were the most commonly reported outcome (*n* = 40), followed by measures of disease burden (*n* = 15). Other reported outcomes included vaccination coverage, disease transmission rates, case detection rates, animal and human productivity traits, weight loss, and animal welfare scores. Most studies described positive (*n* = 43) or partially positive (*n* = 6) non-monetary outcomes, such as reduced number of deaths, decreased prevalence, or increased disability-adjusted life years (DALYs) saved. Three studies reported no significant difference in outcome between the OH intervention and control groups.

**Fig. 5:**
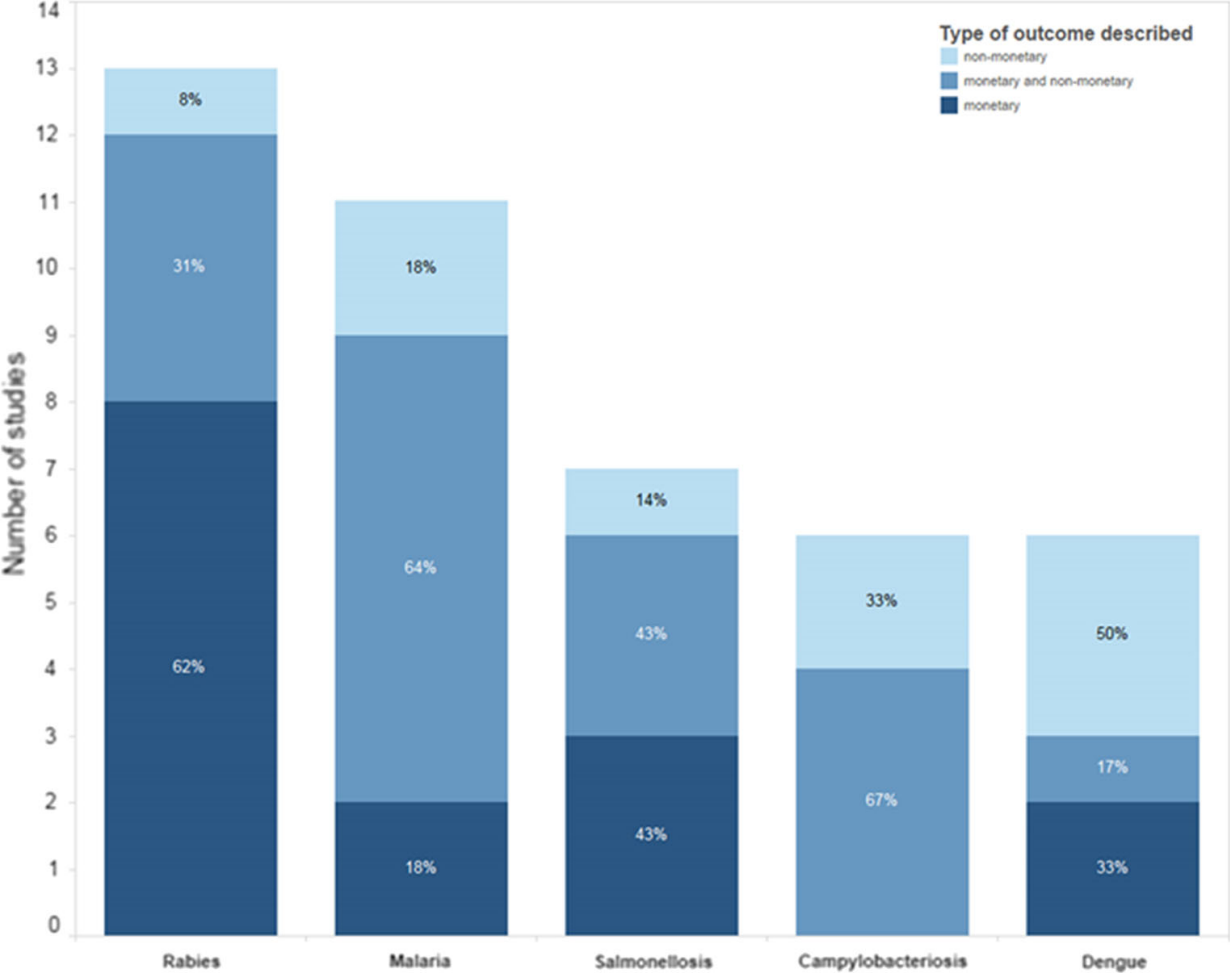
Proportion of studies that described monetary, non-monetary, or both outcomes to assess the top five diseases included in a scoping review on the quantitative outcome of a One Health approach to address complex global health challenges.

The quantitative outcomes reported in studies pertaining to the top five diseases were examined in further detail (Fig. 5). The majority of the rabies studies included in this review showed the benefits, in terms of cost savings or deaths averted, that could be accrued through either dog or wildlife vaccination campaigns (Table 2). The food-borne zoonoses’ studies illustrated the potential reduction in disease primarily via best management practices at the farm and slaughterhouse level (Tables 3 and 4), while the vector-borne studies illustrated benefits in terms of the interventions’ cost-effectiveness or their impact on disease transmission (Tables 5 and 6).

**Table 2:** An Overview of the Type and Value of Quantitative Outcomes Featuring in Those Studies that Described One Health Interventions to Address Rabies Included in this Scoping Review.

References	Geographical location	Intervention	Type of quantitative outcome described	Outcome reported
*Dogs*				
Bögel and Meslin (1990)	Developing countries	Combined dog vaccination and human PEP^a^	Cost efficiency	Cost-efficient in 5 years
Fishbein et al. (1991)	Philippines	One-year dog vaccination campaign	Time to recoup costs	4.1–11.0 years
Fitzpatrick et al. (2014)	Tanzania	Annual dog vaccination campaigns (at different vaccination coverage)	Number of deaths averted	0.6–2.0
			Percentage of deaths averted	8.3–39.3%
			Cost-effectiveness	Cost-effective to very cost-effective
Häsler et al. (2014b)	Sri Lanka	Dog vaccination and other control interventions	DALYs^b^ averted	738
			Animal welfare impact score	Improved
			Program costs	US$ 1.03 million
Pinto et al. (2011)	Brazil	Dog vaccination (vs. human PEP^a^)	Cost comparison	Costs 9.2–20.2 lower (in Brazilian Real)
Tenzin and Ward (2012)	Bhutan	Combined dog vaccination and human PEP^a^ (vs. human PEP^a^ only)	Cost savings	US$ 0.09 million saved after 6 years
Townsend et al. (2013)	Bali	Comprehensive high coverage dog vaccination	Human lives saved over 10 years	550
			Money saved over 10 years	US$ 15 million
Zinsstag et al. (2009)	Chad	One-year dog vaccination campaign	Cost per death averted	US$ 596 by 10th year
			Time to recoup costs	5.9 years
*Wildlife*				
Aubert (1999)	France	Wildlife vaccination (vs. fox depopulation)	Cost–benefit analysis	Beneficial after 4th year
Ministère de la Santé et de la Protection Sociale Française (1989)	France	Evaluation of oral vaccination programs in wildlife	Cost–benefit analysis	Beneficial in 10–12 years (less for some departments)
Shwiff et al. (2011)	Canada	Rabies control program including fox vaccination	Benefit–cost ratio	0.49–1.36
			Cost savings	US$ 35.48–98.41 million
Shwiff et al. (2012)	Canada	Rabies control programs including raccoon vaccination	Benefit–cost ratio	0.96–1.55
			Cost savings	US$ 46.70–52.93 million
Uhaa et al. (1992)	USA	Administration of oral vaccines to raccoons	Benefit–cost ratio	2.21–6.80
			Cost savings	US $1.95 million

^a^*PEP* post-exposure prophylaxis.

^b^*DALYs* disability-adjusted life years.

**Table 3:** An Overview of the Type and Value of Quantitative Outcomes Featured in Those Studies that Described One Health Interventions to Address Salmonellosis Included in this Scoping Review.

References	Geographical location	Intervention	Type of quantitative outcome described	Outcome reported
Goldbach and Alban (2006)	Denmark	Hot water decontamination of pig carcasses	Net present value	3.5 million Euro over 15 years
Kangas et al. (2007)	Finland	Salmonella control policies in broiler production	Benefit–cost ratio	0.04–21.25
Korsgaard et al. (2009)	Denmark	Salmonella control programs in egg production	Number of human cases averted	10,200 (95% CI: 8100–12,400)
			Societal costs saved	23.3 million Euro (95% CI: 16.3– 34.9)
			Cost–benefit ratio	0.5
Miller et al. (2005)	USA	Pig vaccination	Reduction in human cases	60%
			Benefit–cost ratio	Less than 1
		Pig carcass rinsing at various water temperatures	Benefit–cost ratio	Greater than 1
Persson and Jendteg (1992)	England, Wales and Sweden	Use of competitive exclusion in poultry production	Costs of illness saved	Up to 12.6 million GBP
Romero-Barrios et al. (2013)	European Union	Interventions on pig farms and during pig slaughter	Risk reduction	Up to 90% risk reduction
Wegener et al. (2003)	Denmark	Salmonella control programs in pig and poultry production	Costs saved	US $25.5 million

**Table 4:** An Overview of the Type and Value of Quantitative Outcomes Featured in Those Studies that Described One Health Interventions to Address Campylobacteriosis Included in this Scoping Review.

References	Geographical location	Intervention	Type of quantitative outcome described	Outcome reported
Gellynck et al. (2008)	Belgium	Decontamination of poultry carcasses with electrolyzed oxidizing water	Cost–benefit ratio	17.66
		Decontamination of poultry carcasses with lactic acid		4.06
		Phage therapy used on chicken farms		2.54
Havelaar et al. (2007)	The Netherlands	Strict hygienic measures on chicken farms	Cost-effectiveness based on a cost–utility ratio^a^ ≤ Euro 50,000/DALYs^b^	Cost-effective
		Reduced fecal leakage during carcass processing		Cost-effective
		Chemical decontamination of poultry carcasses		Cost-effective
Jensen and Jensen (2013)	European Union	Vaccination of chicks	Cost neutralization	1.65 Euro per vaccine dose
Lake et al. (2013)	New Zealand	Poultry slaughterhouse improvements (e.g., new evisceration machines)	Cost per DALYs^b^ saved	NZ$ 1200
		Continuous chemical treatment of poultry carcass		NZ$ 1700
		Phage-based controls on chicken farms		NZ$ 3000
Mangen et al. (2007)	The Netherlands	Phage therapy used on chicken farms	Cost-effectiveness based on a cost–utility ratio^a^ ≤ 100,000 Euro/DALYs^b^	Cost-effective
Romero-Barrios et al. (2013)	European Union	Application of fly screens in indoor poultry flocks	Risk reduction	60%
		Treating or freezing broiler carcasses		87–98%

^a^Cost–utility ratio is described as the ratio of the net cost of intervention to averted disease burden in DALYs.

^b^*DALYs* disability-adjusted life years.

**Table 5:** An Overview of the Type and Value of Quantitative Outcomes Featuring in Those Studies that Described One Health Interventions to Address Malaria Included in this Scoping Review.

References	Geographical location	Intervention	Type of quantitative outcome described	Outcome reported
Aikins et al. (1998)	Gambia	Use of ITN^a^	Cost-effectiveness per death averted	US$ 471
			Cost-effectiveness per discounted life years gained	US$ 31.53
Akhavan et al. (1999)	Brazil	National malaria control program including vector control	Cost-effectiveness per life saved	US$ 2672
			Cost-effectiveness per DALYs^b^ averted	US$ 69
Gatton and Cheng (2010)	Australia	ITN^a^ and chemotherapy	Disease transmission	No transmission possible
Goodman et al. (1999)	Low-income country in sub-Saharan Africa	Provision of bed nets	Cost-effectiveness per DALYs^b^ averted	US$ 19–85
		Insecticide treatment of existing bed nets		US$ 4–10
Goodman et al. (2001)	South Africa	ITN^a^ (vs. residual house spraying)	Effectiveness (adjusted rate ratio based on number of cases)	0.69
			Cost per case averted	US$ 16
			Cost per death averted	US$ 1696
Mueller et al. (2008)	Togo	Three-year ITN^a^ campaign	Number of deaths averted	6285
			Number of cases averted	1.2 million
			Cost per death averted	US$ 635
			Cost per DALYs^b^ averted	US$ 16.39
Mulligan et al. (2008)	Tanzania	ITN^a^ voucher program	Number of child deaths averted	12,039
			Cost per child death averted	US$ 873
Pulkki-Brännström et al. (2012)	Not specified	Long-lasting ITN^a^ (vs. conventional ITN^a^)	Child deaths averted	30,800
			DALYs^b^ averted	1.02 million
			Cost per DALYs^b^ averted	US$ 16.8
			Cost-effectiveness	Cost-effective if priced at no more than US$ 1.5 above conventional ITN^a^
Riedel et al. (2010)	Zambia	Bed nets	Odds of parasitaemia	40% less (12–60%)
Smithuis et al. (2013)	Myanmar	ITN^a^ (vs. early diagnosis and effective treatment)	Cost per DALYs^b^ averted	US$ 51
Yhdego and Majura (1988)	Tanzania	Comparison of two vector control programs: engineering vs. use of larvicides and insecticides	Program effectiveness	97 vs. 75%
			Cost-effectiveness	Tshs 2.8 million vs. Tshs 10.5 million

^a^*ITN* insecticide-treated bed nets.

^b^*DALYs* disability-adjusted life years.

**Table 6:** An Overview of the Type and Value of Quantitative Outcomes Featured in Those Studies that Described One Health Interventions to Address Dengue Included in this Scoping Review.

References	Geographical location	Intervention	Quantitative outcome described	Values reported
Díaz (2012)	Cuba	Integrated surveillance system	Detection of febrile cases	Increased
McConnell and Gubler (2003)	Puerto Rico	Control of vector breeding sites	Cost-effectiveness	Cost-effective if dengue transmission is reduced by 50% and intervention costs less than US$ 2.50 per person
Ocampoa et al. (2014)	Colombia	Identification and spraying of vector breeding sites	Rate ratio of human incidence	0.19 (95% CI 0.12–0.30) compared to control area
Orellano and Pedroni (2008)	Argentina	Fumigation of vectors	Net present value	I$ 196,879
			Cost–benefit analysis	Beneficial when more than 1363 cases of dengue and at least 1 case of dengue hemorrhagic fever are averted
Suaya et al. (2007)	Cambodia	Annual targeted larvicidal campaigns	Cost per DALYs^a^ saved (public perspective)	US$ 313
			Cost per DALYs^a^ saved (societal perspective)	US$ 37
Tsunoda et al. (2013)	Vietnam	Use of insecticide-treated nets to cover water reservoirs	Human seroprevalence	62.2% (vs. 74.6% in control area)
		Addition of insecticide to other water containers		

^a^*DALYs* disability-adjusted life years.

### Quality Assessment

To perform a quality assessment on the included studies, judgement was made as to whether the methods were explicitly stated. The majority of the studies (*n* = 69) were determined to have clearly explained and reproducible methods, while six studies lacked certain information and were therefore considered as partly reproducible. For the remaining ten studies, the methods were considered insufficiently described; there were no recognizable similarities between these studies as they were conducted in different regions and described different health issues (S7–S12).

## Discussion

This study provides an extensive evidence base for research highlighting the quantitative outcomes, both monetary and non-monetary, of an OH approach. Moreover, it adds to recently published reviews (Häsler et al. 2014a; Baum et al. 2017) by also including research that may not have explicitly included definitions or terminology relating to “One Health” but employed a OH approach. This work is of substantial importance in relation to decision-making at the policy or governmental level and provides some proof that financing OH projects can be beneficial in a number of ways. Additionally, this review showcases the approaches used by a number of researchers and organizations that could be utilized in a number of global economic settings to improve human and animal health and welfare.

Most of the included studies dealt with biotic health issues, and the top five diseases were rabies, malaria, salmonellosis, campylobacteriosis, and dengue; this could be driven by funding priorities which are often focused on large global health challenges. Three of these are zoonoses, while the other two are vector-borne diseases. It is not surprising that zoonoses would be among the most commonly addressed OH topics as they are suited for a collaborative approach between human and veterinary medicine, such as through joint human–animal vaccination programs, integrated surveillance, and increased investment in cost-effective animal-level interventions with consequent human health benefits (Roth et al. 2003; Schelling et al. 2007; Zinsstag et al. 2009; Tschopp et al. 2013, Stärk et al. 2015).

Rabies is a clear example where OH approaches can be beneficial. Thirteen of the included studies described rabies, and all investigated vaccination as an option of controlling rabies in either dogs or wildlife. Most of these studies showed that those control programs that include vaccination are often cost-effective over a long time span, ranging from 4.1 to 11.0 years in the Philippines (Fishbein et al. 1991), 5.9 years in N’Djaména (Zinsstag et al. 2009), and 6 years in Bhutan (Tenzin and Ward 2012).

Our review also identified several OH interventions targeting food-borne zoonoses, a growing concern due to the increased demand for livestock products and consequent intensification and globalization of the food market (Karesh et al. 2012; Wall 2014). The importance of food safety for the general public and policy-makers was emphasized in a recent document by the European Union Scientific Steering Committee (European Union Scientific Steering Committee 2015) and was reiterated in the choice of Food Safety as the topic for the 2015 World Health Day (Chan 2014). Seven studies described interventions to control salmonellosis in either poultry or pig production systems, and considered the effect of these interventions on the number of human cases and overall costs incurred. Competitive exclusion (Persson and Jendteg 1992), control programs (Kangas et al. 2007; Korsgaard et al. 2009), and management practices such as hot water decontamination of carcasses (Miller et al. 2005; Goldbach and Alban 2006) were all found to be economically effective interventions. Similarly, the other benefits listed for *Salmonella* and other food-borne diseases such as *Campylobacter* could be utilized by policy-makers to keep these diseases to a minimum.

Vector-borne diseases, such as malaria and dengue, also featured prominently in our list of included studies. All the malaria studies assessed control programs which included vector control, mostly through the use of insecticide-treated bed nets (ITNs). In several African countries, ITNs (and long-lasting ITNs) proved to be effective in reducing the disease (Goodman et al. 1999; Riedel et al. 2010), though these benefits were sometimes outweighed by the costs incurred (Goodman et al. 2001; Pulkki-Brännström et al. 2012). The WHO recommends only distributing long-lasting ITNs (World Health Organization 2007); the findings in the current study are valuable in identifying those interventions that are superior to others when a number are available. These studies also emphasize the importance of environmental interventions, such as vector control, improved sanitation and hygiene, and integrated surveillance programs, to control the human impact of such diseases (World Health Organization 2014). Increased trade and globalization, together with climate change, habitat encroachment, and forest fragmentation, have augmented the possibility of vector-borne disease transmission (Sherman 2010), and this was exemplified by the recent emergence of Chikungunya and Zika virus in Latin America and the Caribbean (World Health Organization 2016a). Cross-sectorial approaches identified in this review could therefore set an example for future endeavors focusing on emerging vector-borne diseases. Ultimately it appears that the magnitude of benefit and the timescale over which control programs must be in place for the realization of benefit is disease and environment dependent. There is value in policy-makers identifying diseases and contexts similar to their own within this review to use as framework for designing programs specific to their own situations.

While the top biotic health issues described in our included studies may reflect funding priorities, they also mirror to a large extent recent findings on the global burden of disease (GBD). Infectious diseases such as rabies, malaria, and dengue are ranked among the top six WHO parasitic and vector-borne diseases (World Health Organization 2016b), and among the top ten NTD by the Lancet (Global Burden Disease 2015 DALYs and HALE Collaborators, 2016). Similarly, among all food-borne hazards, campylobacteriosis and salmonellosis, together with enteropathogenic *Escherichia coli*, were found to be the most relevant contributors to DALYs (World Health Organization Global Burden of Foodborne Diseases 2015). Noticeably, other zoonotic diseases with a high GBD, such as leishmaniasis or schistosomiasis, rarely featured in our findings. Reasons for this might be either that the OH interventions have not yet been used for their control, or that the study outcome was not assessed in a quantitative manner or it could not be attributed clearly to the OH intervention. Recent guidelines for OH studies, which also encourage authors to mention how they think the OH approach added value to the study, should help by clarifying whether a OH approach was used in the study and how it contributed to the final outcome (Davis et al. 2017).

In our review, abiotic health issues, such as respiratory disease due to air pollution or metal intoxication, were only described in 16.5% of the included studies. The importance of considering the environmental component of public health was recently reiterated in the Hanoi Declaration (Hanoi Declaration 2015) and subsequent Sustainable Development Goals [particularly non-communicable conditions such as cardiac disease, cancer, and obesity (United Nations 2015)]. Therefore, these cross-sectorial studies that tackle abiotic health issues, such as the impact of air and water pollution on human health, bring to light opportunities and avenues for a collaborative OH approach which need not be limited to communicable diseases. Two studies included in this review investigated the positive health benefits accrued through dog walking (Bauman et al. 2001; Kushner et al. 2006). Dog ownership encourages owner physical activity and has been described as a cost-effective and socially acceptable preventive measure for the current obesity epidemic (Mills and Hall 2014). This highlights the opportunity for improved disease prevention and control through OH approaches, by investigating the pivotal human–animal companionship relationship to combat not only obesity, but also depression and cognitive disorders.

Antimicrobial resistance (AMR) did not feature in any of our included studies. This was surprising given both the attention it has received in recent years, and its complex and multifaceted nature which makes it amenable to cross-sectorial approaches (Queenan et al. 2016; Singh 2017; World Health Organization 2017). Since our literature search was conducted in 2014, it is likely the more recent focus on AMR in published research in the last few years would not have been captured. Similarly, we may have missed studies that describe a OH approach when dealing with other health issues, such as salmonellosis and trypanosomiasis, but were published after our final literature search was conducted (Sundström et al. 2014; Shaw et al. 2015).

The majority of the 85 studies included for qualitative synthesis were performed after 2000. This is not surprising as the OH initiative has been gaining momentum over the past decade, and the amount of interdisciplinary research has been shown to be increasing (Stärk et al. 2015; Van Noorden 2015). Nonetheless, segregation between disciplines still persists, particularly between the veterinary and ecological sciences (Manlove et al. 2016), and future interdisciplinary studies should ensure that the ecosystem component is properly represented (Barrett and Bouley 2015). Most identified studies described modeling approaches, either as mathematical modeling of infectious diseases or economic analyses. We realize that this may have been biased both by our search terms which targeted such studies and by our inclusion criteria which selected only for those studies that had a quantitative outcome. However, we think that this could also be partly due to the fact that some of the topics addressed may be hard to implement in the field given their underlying complexity. Moreover, funding for such interdisciplinary endeavors may be hard to obtain, thus making modeling approaches a more feasible and economically viable option.

One of the greatest challenges of this review lay with the definition of OH. The definition provided by the American Veterinary Medical Association (2008) was chosen to inform the review, and several examples were provided within the screening forms to ensure consistency in the interpretation of OH. Despite this, the interpretation of some references was difficult. Therefore, it is possible that studies may have been excluded which according to other definitions may be considered OH or, conversely, included studies which may not be considered OH. The recently published COHERE checklist for OH studies (Davis et al. 2017) should help with such future endeavors by setting a benchmark as to what should be considered a OH approach.

The final list of studies only included around 0.0025% of all screened references. This was expected given the broad search terms used. It was agreed that given the objective to identify those studies that described a OH approach (without necessarily containing the term OH), the sensitivity of the search should be prioritized over the specificity. Despite the broad search terms, a certain publication bias is to be expected based on the selection of literature databases, although they were selected pertinent to the type of studies that were sought in the review. An information specialist who specializes in objective, structured reviews of the literature (DG) was consulted and involved in the process of this review to ensure that the most appropriate databases were searched. Furthermore, we attempted to identify relevant studies in the gray literature through our search verification, which included expert elicitation and review of relevant textbooks. Future work should prioritize investigating these alternative sources further, as it is possible that the expected positive publication bias could have affected the results obtained.

As our review question focused on quantitative outcomes, we excluded those studies which described qualitative outcomes of a OH approach, such as improved knowledge on health topics, changes in attitude or practices, or improved participation, which are a necessary preceding step to ensure uptake and implementation of interventions and practices (World Health Organization 2014). These outcomes may be harder to evaluate as they are often intangible and incommensurable. Yet they are important components of the overall societal benefit and should therefore be taken into consideration when making decisions regarding fund allocation for disease control programs or other interventions.

We note that during the full-text screening process we excluded 60 references which described a OH approach but not a quantitative outcome. This lack of reported outcomes is similar to findings reported by other recently published reviews (Häsler et al. 2014a; Baum et al. 2017) and underlines a gap in current published research, where missing quantification of the evidence may hinder the uptake of research findings. Additionally, while this review identified a numerous diversity of monetary and non-monetary terms, this diversity in itself may impede comparisons between studies. We therefore encourage harmonization of metrics to ensure that future research is both outcome-based and comparable, thus facilitating interpretation and implementation of findings based on OH approaches. It is important for a number of stakeholders to be involved in the decision-making process in relation to the prioritization of which outcomes should be consistently measured in studies employing a OH approach. All levels of decision-makers should be included in the process, from those in the field to those at the policy-making level. This will ensure that the most appropriate outcomes, and therefore the most likely to be successfully captured, are identified. It is suggested that structured objective frameworks such as the Delphi methodology (Okoli and Pawlowski 2004) and those employed by the James Lind Alliance (http://www.jla.nihr.ac.uk/) be utilized for this purpose.

This review identifies a number of studies that may not have included terminology relating to OH but have employed a OH approach. Additionally, this is the first time that the quantitative outcomes of OH studies have been collectively reported, and therefore could provide an additional resource for policy-makers to utilize for similar OH research studies in the future. Future work should focus on investigating further the gray literature for other similar studies and the harmonization of metrics employed to determine the success of approaches across all OH studies.

## Electronic supplementary material

Below is the link to the electronic supplementary material.

Supplementary material 1 (DOCX 68 kb)
