# Supplementary material for: Quantitative Outcomes of a One Health approach to Study Global Health Challenges
Source: Ecohealth. 2018 Jan 12;15(1):209–27. doi: 10.1007/s10393-017-1310-5 (PMC6003973; doi:10.1007/s10393-017-1310-5)
Supplement: Supplementary file 1 — Supplementary material 1 (DOCX 68 kb) [file 10393_2017_1310_MOESM1_ESM.docx]

**S1.** Relevance tool for title and abstract screening in a scoping review on the quantitative outcome when using a One Health approach to address complex global health challenges

On studies that describe a quantitative outcome (in monetary and/or non-monetary terms) of a “One Health” approach

1. Does this abstract investigate primary reseach^1^?

- Yes (include)
- Literature Review (exclude)
- Other^2^ (exclude)

^1^ Primary research represents a study where the author(s) collected and analyzed their own data. Literature reviews, commentaries essay papers are excluded

^2^ Other studies can be commentaries, letters to the editor, etc.

2. Does this abstract describe a Quantitative Benefit^3^ of using a “One Health” approach^4^?

- Yes (include)
- No (exclude)

^3^ **Quantitative Benefit** may be defined as the monetary (e.g. cost analysis, cost benefit, opportunity cost) and non-monetary (e.g. DALYs, HALYs, QALYs, societal benefit) positive outcome/profit of an efficient resource allocation, associated with the protection of the environment, higher quality and/or quantity of data collection, improvement in human health and well-being, improvement in animal health and welfare, and early detection and timely response to future threats.

^4^ **One Health** approach is defined as “the collaborative efforts of multiple disciplines working locally, nationally and globally to attain optimal health for people, animals and our environment” (American Veterinary Medical Association, 2008)

3. Was this study conducted in or after 1910?

- - Yes (include)
  - No (exclude)

4. What language is the abstract written in?

- - English, German, Italian, French, Portuguese, Greek, Dutch, Finnish, Russian, Norwegian, Swedish (include)
  - Other language (exclude)

**S2.** Relevance tool for full-text screening in a scoping review on the quantitative outcome when using a One Health approach to address complex global health challenges

On studies that describe a quantitative outcome (in monetary and/or non-monetary terms) of a “One Health” approach to study complex health issues.

1. Does this full-text article investigate primary reseach^1^?

- Yes (include)
- Literature Review (exclude)
- Other^2^ (exclude)

^1^ Primary research represents a study where the author(s) collected and/or analyzed data. These include:

- Case report/case series
- Qualitative studies
- Observational studies: cross-sectional, cohort and case-control studies
- Experimental studies: randomized clinical trials, controlled trials, and challenge trials
- Mathematical models and economic studies based on field data (collected in the same study or elsewhere)

^2^ Other studies can be Commentaries, Letters to the editor, etc. These should be excluded.

2. Does this full-text article describe a “One Health”^3^ approach?

- Yes (include)
- No (exclude)

^3^ **One Health** approach is defined as “the collaborative efforts of multiple disciplines working locally, nationally and globally to attain optimal health for people, animals and our environment” (American Veterinary Medical Association, 2008)

Examples of “One Health approaches” include, but are not limited to:

- Food safety e.g. interventions at the food chain with consequent reduction of human disease;
- Human-animal bond e.g. pet therapy; illustrations of how keeping animals may improve human health or reduce obesity, etc.
- Zoonotic vector-borne pathogens e.g. impacting vector biology to reduce disease incidence (include malaria, dengue, Schistosomiasis, Rift Valley Fever etc.)
- Emerging infectious diseases e.g. interventions at animal or environmental level to reduce antimicrobial resistance in humans
- Zoonotic diseases e.g. vaccination in dogs to reduce rabies incidence in humans: combining animal and human vaccination programs to make them more cost-efficient
- Animal or human level interventions which impact environmental health e.g. reduction in cow methane production; aquaculture systems affecting water pollution

To be considered as “One Health”, it must describe an animal-human, animal-environment, human-environment, or animal-human-environment interaction, but not necessarily describe a benefit for both (e.g. could be an intervention at the animal level, with a benefit on the human level).

Our “One Health” definition will not include:

- Food security e.g. improving animal nutrition or animal genetics to improve sustainability of livestock therefore alleviating poverty
- Comparative anatomy e.g. using dogs/cats to study pathogenesis of human diseases such as lymphoma or rheumatoid arthritis

3. Does this full-text article describe a Quantitative Outcome^4^ at the animal, human, or environmental health level?

- Yes (include)
- No (exclude)

^4^ **Quantitative Outcome** may be defined as the monetary (e.g. cost analysis, cost benefit, opportunity cost) and non-monetary (e.g. DALYs, HALYs, QALYs, societal benefit, reduced disease incidence, prevalence, morbidity, mortality or case-fatality rate) outcome of an efficient resource allocation, associated with the protection of the environment, higher quality and/or quantity of data collection, improvement in human health and well-being, improvement in animal health and welfare, and early detection and timely response to future threats.

4. What language is the full-text written in?

- - English, German, Italian, French, Portuguese, Greek, Dutch, Finnish, Russian, Norwegian, Swedish, Spanish (include)
  - Other language (exclude)

**S3.** The search strategy used in CAB Abstracts to identify studies that described a quantitative outcome when using a One Health approach to address complex global health challenges (mp=keywords; /=subject heading)

Database: CAB Abstracts <1910 to 2014 Week 21>

Search Strategy:

--------------------------------------------------------------------------------

1 (animal and human).mp. [mp=abstract, title, original title, broad terms, heading words] (88433)

2 (animals and human).mp. [mp=abstract, title, original title, broad terms, heading words] (614548)

3 (animal and humans).mp. [mp=abstract, title, original title, broad terms, heading words] (20676)

4 (animals and humans).mp. [mp=abstract, title, original title, broad terms, heading words] (64583)

5 (human and environment).mp. [mp=abstract, title, original title, broad terms, heading words] (32398)

6 (humans and environment).mp. [mp=abstract, title, original title, broad terms, heading words] (5382)

7 (animal and environment).mp. [mp=abstract, title, original title, broad terms, heading words] (28411)

8 (animals and environment).mp. [mp=abstract, title, original title, broad terms, heading words] (110589)

9 animal to human.mp. (3596)

10 human to animal.mp. (9189)

11 social-ecological.mp. (902)

12 socio-ecological.mp. (552)

13 One Health.mp. (429)

14 Ecohealth.mp. (51)

15 One World.mp. (96)

16 One Medicine.mp. (69)

17 (ecosystem and health).mp. [mp=abstract, title, original title, broad terms, heading words] (4238)

18 (holistic and health).mp. [mp=abstract, title, original title, broad terms, heading words] (1004)

19 (veterinary and human medicine).mp. [mp=abstract, title, original title, broad terms, heading words] (862)

20 interdisciplinary.mp. or exp interdisciplinary research/ (6169)

21 multidisciplinary.mp. (5928)

22 transdisciplinary.mp. (476)

23 cross sector.mp. (117)

24 inter sector.mp. (73)

25 trans sector.mp. (4)

26 zoonoses.mp. or exp zoonoses/ (31860)

27 zoonosis.mp. (2979)

28 zoonotic.mp. (34514)

29 veterinary public health.mp. (633)

30 VPH.mp. (53)

31 farm to fork.mp. (306)

32 stable to table.mp. (44)

33 value chain.mp. (1391)

34 1 or 2 or 3 or 4 or 5 or 6 or 7 or 8 or 9 or 10 or 11 or 12 or 13 or 14 or 15 or 16 or 17 or 18 or 19 or 20 or 21 or 22 or 23 or 24 or 25 or 26 or 27 or 28 or 29 or 30 or

31 or 32 or 33 (791696)

35 DALY*.mp. (692)

36 HALY*.mp. (473)

37 QALY*.mp. (333)

38 disability adjusted life year.mp. (129)

39 disability adjusted life years.mp. (355)

40 health adjusted life year.mp. (0)

41 health adjusted life years.mp. (1)

42 quality adjusted life year.mp. (104)

43 quality adjusted life years.mp. (136)

44 expected quality adjusted life year.mp. (0)

45 expected quality adjusted life years.mp. (5)

46 opportunity cost.mp. (1252)

47 opportunity costs.mp. or exp opportunity costs/ (1487)

48 cost benefit.mp. (19605) 49 cost benefits.mp. (338)

50 cost analys*.mp. (6378)

51 exp "cost analysis"/ (4642)

52 cost assessment.mp. (105)

53 cost effectiveness.mp. (6798)

54 exp "cost effectiveness analysis"/ (2440)

55 cost utility.mp. (108)

56 cost utilities.mp. (0)

57 profit*.mp. (72129)

58 exp profits/ (6277)

59 cost allocation.mp. (108)

60 cost benefit analys*.mp. [mp=abstract, title, original title, broad terms, heading

words] (17794)

61 exp "cost benefit analysis"/ (16400)

62 exp "cost control"/ or cost control.mp. (545)

63 cost controls.mp. (15)

64 cost saving.mp. (888)

65 cost savings.mp. (1756)

66 costs savings.mp. (64)

67 cost of illness.mp. (170)

68 costs of illness.mp. (49)

69 cost of disease.mp. (136)

70 costs of disease.mp. (87)

71 cost of intervention.mp. (169)

72 costs of intervention.mp. (39)

73 cost sharing.mp. (420)

74 costs sharing.mp. (3)

75 health care cost.mp. (85)

76 exp health care costs/ or health care costs.mp. (2901)

77 health care expenditure.mp. (93)

78 health care expenditures.mp. (87)

79 value of life.mp. (68)

80 societal benefit.mp. (23)

81 economic evaluation.mp. or exp economic evaluation/ (8957)

82 economic analys*.mp. (23908)

83 exp economic analysis/ (46830)

84 economic assessment.mp. (1373)

85 health economics.mp. (207)

86 resource allocation.mp. or exp resource allocation/ (5140)

87 cost avoidance.mp. (10)

88 costs avoidance.mp. (0)

89 loss avoidance.mp. (6)

90 losses avoidance.mp. (0)

91 35 or 36 or 37 or 38 or 39 or 40 or 41 or 42 or 43 or 44 or 45 or 46 or 47 or 48

or 49 or 50 or 51 or 52 or 53 or 54 or 55 or 56 or 57 or 58 or 59 or 60 or 61 or 62 or

63 or 64 or 65 or 66 or 67 or 68 or 69 or 70 or 71 or 72 or 73 or 74 or 75 or 76 or 77

or 78 or 79 or 80 or 81 or 82 or 83 or 84 or 85 or 86 or 87 or 88 or 89 or 90 (142468)

92 34 and 91 (9660)

***************************

**S4.** The search strategy used for the National Health Service Economic Evaluation Database (part of the NIHR Centre for Reviews and Dissemination; <http://www.crd.york.ac.uk/CRDWeb/ResultsPage.asp>) to identify studies that describe a quantitative outcome when using a One Health approach to address complex global health challenges

(animal and human) OR (animals and human) OR (animal and humans) OR (animals and humans) OR (human and environment) OR (humans and environment) OR (animal and environment) OR (animals and environment) OR animal to human OR human to animal OR social-ecological OR socio-ecological OR One health OR ecohealth OR one world OR one medicine OR (ecosystem and health) OR (holistic and health) OR (veterinary and human medicine) OR interdisciplinary OR multidisciplinary OR transdisciplinary OR cross sector OR inter sector OR trans sector OR zoonosis OR zoonoses OR zoonotic OR veterinary public health OR VPH OR farm to fork OR stable to table OR value chain

OR

cats OR dogs OR cattle OR horses OR swine OR sheep OR chickens OR rabbits OR wildlife OR bird OR birds OR animal OR animals OR veterinary

Rationale behind search:

This database only contains literature relating to humans, therefore the idea was to search for animal related studies only.

The original search is as per our plan, the animal terms were added in to ensure that any other papers could be picked up. The animal terms used are MeSH terms in Medline (Embase uses the same MeSH terms as Medline), plus some which needed to be added in (wildlife, bird, birds, animal, animals, veterinary)

**S5.** The search strategy used in MEDLINE to identify studies that describe a quantitative outcome when using a One Health approach to address complex global health challenges. mp=keywords; / = subject heading

Database: Ovid MEDLINE(R) In-Process & Other Non-Indexed Citations and Ovid

MEDLINE(R) <1946 to Present>

Search Strategy:

--------------------------------------------------------------------------------

1 (animal and human).mp. [mp=title, abstract, original title, name of substance word, subject heading word, keyword heading word, protocol supplementary concept word, rare disease supplementary concept word, unique identifier] (150473)

2 (animals and human).mp. [mp=title, abstract, original title, name of substance word, subject heading word, keyword heading word, protocol supplementary concept word, rare disease supplementary concept word, unique identifier] (737887)

3 (animal and humans).mp. [mp=title, abstract, original title, name of substance word, subject heading word, keyword heading word, protocol supplementary concept word, rare disease supplementary concept word, unique identifier] (242182)

4 (animals and humans).mp. [mp=title, abstract, original title, name of substance word, subject heading word, keyword heading word, protocol supplementary concept word, rare disease supplementary concept word, unique identifier] (1537792)

5 (human and environment).mp. [mp=title, abstract, original title, name of substance word, subject heading word, keyword heading word, protocol supplementary concept word, rare disease supplementary concept word, unique identifier] (48329)

6 (humans and environment).mp. [mp=title, abstract, original title, name of substance word, subject heading word, keyword heading word, protocol supplementary concept word, rare disease supplementary concept word, unique identifier] (201510)

7 (animal and environment).mp. [mp=title, abstract, original title, name of substance word, subject heading word, keyword heading word, protocol supplementary concept word, rare disease supplementary concept word, unique identifier] (26680)

8 (animals and environment).mp. [mp=title, abstract, original title, name of substance word, subject heading word, keyword heading word, protocol supplementary concept word, rare disease supplementary concept word, unique identifier] (101926)

9 animal to human.mp. (7933)

10 human to animal.mp. (11400)

11 exp Social Environment/ or social-ecological.mp. (89882)

12 socio-ecological.mp. (284)

13 One Health.mp. (1320)

14 Ecohealth.mp. (60)

15 One World.mp. (129)

16 One Medicine.mp. (126)

17 (ecosystem and health).mp. [mp=title, abstract, original title, name of substance word, subject heading word, keyword heading word, protocol supplementary concept word, rare disease supplementary concept word, unique identifier] (3611)

18 (holistic and health).mp. [mp=title, abstract, original title, name of substance word, subject heading word, keyword heading word, protocol supplementary concept word, rare disease supplementary concept word, unique identifier] (11783)

19 (veterinary and human medicine).mp. [mp=title, abstract, original title, name of substance word, subject heading word, keyword heading word, protocol supplementary concept word, rare disease supplementary concept word, unique identifier] (594)

20 interdisciplinary.mp. (30488)

21 multidisciplinary.mp. (44476)

22 transdisciplinary.mp. (790)

23 cross sector.mp. (112)

24 inter sector.mp. (46)

25 trans sector.mp. (2)

26 zoonoses.mp. or exp Zoonoses/ (12846)

27 zoonosis.mp. (2801)

28 zoonotic.mp. (9045)

29 veterinary public health.mp. (343)

30 VPH.mp. (174)

31 farm to fork.mp. (115)

32 stable to table.mp. (16)

33 value chain.mp. (198)

34 1 or 2 or 3 or 4 or 5 or 6 or 7 or 8 or 9 or 10 or 11 or 12 or 13 or 14 or 15 or 16or 17 or 18 or 19 or 20 or 21 or 22 or 23 or 24 or 25 or 26 or 27 or 28 or 29 or 30 or 31 or 32 or 33 (2117208)

35 DALY*.mp. (1331)

36 HALY*.mp. (350)

37 QALY*.mp. (5285)

38 disability adjusted life year.mp. (320)

39 disability adjusted life years.mp. (1016)

40 health adjusted life year.mp. (1)

41 health adjusted life years.mp. (9)

42 quality adjusted life year.mp. (2836)

43 quality adjusted life years.mp. or exp Quality-Adjusted Life Years/ (8578)

44 expected quality adjusted life year.mp. (0)

45 expected quality adjusted life years.mp. (28)

46 opportunity cost.mp. (459)

47 opportunity costs.mp. (536)

48 cost benefit.mp. (63931)

49 cost benefits.mp. (641)

50 cost analys*.mp. [mp=title, abstract, original title, name of substance word, subject heading word, keyword heading word, protocol supplementary concept word, rare disease supplementary concept word, unique identifier] (44568)

51 exp "Costs and Cost Analysis"/ (180613)

52 cost assessment.mp. (162)

53 cost effectiveness.mp. (35361)

54 cost utility.mp. (2507)

55 cost utilities.mp. (13)

56 profit*.mp. (17437)

57 cost allocation.mp. or exp "Cost Allocation"/ (1998)

58 cost benefit analys*.mp. [mp=title, abstract, original title, name of substance word, subject heading word, keyword heading word, protocol supplementary concept word, rare disease supplementary concept word, unique identifier] (61253)

59 exp Cost-Benefit Analysis/ (59907)

60 cost control.mp. or exp "Cost Control"/ (29369)

61 cost controls.mp. (127)

62 cost saving.mp. (3749)

63 cost savings.mp. or exp "Cost Savings"/ (15294)

64 costs savings.mp. (121)

65 cost of illness.mp. or exp "Cost of Illness"/ (18016)

66 costs of illness.mp. (194)

67 cost of disease.mp. (173)

68 costs of disease.mp. (183)

69 cost of intervention.mp. (836)

70 costs of intervention.mp. (99)

71 cost sharing.mp. or exp "Cost Sharing"/ (4092)

72 costs sharing.mp. (1)

73 health care cost.mp. (1379)

74 health care costs.mp. or exp Health Care Costs/ (51966)

75 health care expenditure.mp. (628)

76 exp Health Expenditures/ or health care expenditures.mp. (16390)

77 value of life.mp. or exp "Value of Life"/ (6117)

78 societal benefit*.mp. (282)

79 economic evaluation.mp. (5275)

80 economic analys*.mp. [mp=title, abstract, original title, name of substance word, subject heading word, keyword heading word, protocol supplementary concept word, rare disease supplementary concept word, unique identifier] (4025)

81 economic assessment.mp. (500)

82 exp Economics, Medical/ or health economics.mp. (15470)

83 resource allocation.mp. or exp Resource Allocation/ (18896)

84 cost avoidance.mp. (224)

85 costs avoidance.mp. (8)

86 loss avoidance.mp. (35)

87 losses avoidance.mp. (1)

88 35 or 36 or 37 or 38 or 39 or 40 or 41 or 42 or 43 or 44 or 45 or 46 or 47 or 48 or 49 or 50 or 51 or 52 or 53 or 54 or 55 or 56 or 57 or 58 or 59 or 60 or 61 or 62 or 63 or 64 or 65 or 66 or 67 or 68 or 69 or 70 or 71 or 72 or 73 or 74 or 75 or 76 or 77 or 78 or 79 or 80 or 81 or 82 or 83 or 84 or 85 or 86 or 87 (260429)

89 34 and 88 (14053)

***************************

**S6.** The search strategy used in Embase to identify studies that describe a quantitative outcome when using a One Health approach to address complex global health challenges mp=keywords; /=subject heading

Database: Embase <1980 to 2014 Week 22>

Search Strategy:

--------------------------------------------------------------------------------

1 (animal and human).mp. [mp=title, abstract, subject headings, heading word, drug trade name, original title, device manufacturer, drug manufacturer, device trade name, keyword] (1031446)

2 (animals and human).mp. [mp=title, abstract, subject headings, heading word, drug trade name, original title, device manufacturer, drug manufacturer, device trade name, keyword] (138622)

3 (animal and humans).mp. [mp=title, abstract, subject headings, heading word, drug trade name, original title, device manufacturer, drug manufacturer, device trade name, keyword] (120313)

4 (animals and humans).mp. [mp=title, abstract, subject headings, heading word, drug trade name, original title, device manufacturer, drug manufacturer, device trade name, keyword] (53734)

5 (human and environment).mp. [mp=title, abstract, subject headings, heading word, drug trade name, original title, device manufacturer, drug manufacturer, device trade name, keyword] (231358)

6 (humans and environment).mp. [mp=title, abstract, subject headings, heading word, drug trade name, original title, device manufacturer, drug manufacturer, device trade name, keyword] (12201)

7 (animal and environment).mp. [mp=title, abstract, subject headings, heading word, drug trade name, original title, device manufacturer, drug manufacturer, device trade name, keyword] (80413)

8 (animals and environment).mp. [mp=title, abstract, subject headings, heading word, drug trade name, original title, device manufacturer, drug manufacturer, device trade name, keyword] (18451)

9 animal to human.mp. (9140)

10 human to animal.mp. (15288)

11 socio-ecological.mp. (354)

12 One Health.mp. (1517)

13 Ecohealth.mp. (186)

14 One World.mp. (154)

15 One Medicine.mp. (170)

16 (ecosystem and health).mp. [mp=title, abstract, subject headings, heading word, drug trade name, original title, device manufacturer, drug manufacturer, device trade name, keyword] (5086)

17 (holistic and health).mp. [mp=title, abstract, subject headings, heading word, drug trade name, original title, device manufacturer, drug manufacturer, device trade name, keyword] (8551)

18 (veterinary and human medicine).mp. [mp=title, abstract, subject headings, heading word, drug trade name, original title, device manufacturer, drug manufacturer, device trade name, keyword] (662)

19 interdisciplinary.mp. or exp interdisciplinary research/ (35863)

20 multidisciplinary.mp. (64143)

21 transdisciplinary.mp. (985)

22 cross sector.mp. (140)

23 inter sector.mp. (56)

24 trans sector.mp. (1)

25 zoonosis.mp. or exp zoonosis/ (97139)

26 zoonoses.mp. (2805)

27 zoonotic.mp. (10052)

28 veterinary public health.mp. (367)

29 VPH.mp. (254)

30 farm to fork.mp. (131)

31 stable to table.mp. (63)

32 value chain.mp. (292)

33 DALY*.mp. (1693)

34 HALY*.mp. (373)

35 QALY*.mp. (8696)

36 disability adjusted life year.mp. (520)

37 disability adjusted life years.mp. (1224)

38 health adjusted life year.mp. (5)

39 health adjusted life years.mp. (10)

40 quality adjusted life year.mp. or exp quality adjusted life year/ (12881)

41 quality adjusted life years.mp. (5235)

42 expected quality adjusted life year.mp. (1)

43 expected quality adjusted life years.mp. (32)

44 opportunity cost.mp. (548)

45 opportunity costs.mp. (596)

46 cost benefit.mp. (67802)

47 cost benefits.mp. (924)

48 cost analys*.mp. (8040)

49 cost assessment.mp. (242)

50 cost effectiveness.mp. or exp "cost effectiveness analysis"/ (111865)

51 cost utility.mp. or exp "cost utility analysis"/ (6707)

52 cost utilities.mp. (17)

53 profit*.mp. or exp profit/ (48834)

54 cost allocation.mp. (128)

55 exp "cost benefit analysis"/ or cost benefit analys*.mp. (65310)

56 cost control.mp. or exp "cost control"/ (49080)

57 cost controls.mp. (138)

58 cost saving.mp. (5756)

59 cost savings.mp. (11489)

60 costs savings.mp. (205)

61 cost of illness.mp. or exp "cost of illness"/ (14905)

62 costs of illness.mp. (261)

63 cost of disease.mp. (299)

64 costs of disease.mp. (274)

65 cost of intervention.mp. (1074)

66 costs of intervention.mp. (128)

67 cost sharing.mp. (1264)

68 costs sharing.mp. (2)

69 health care cost.mp. or exp "health care cost"/ (203106)

70 health care costs.mp. (11278)

71 health care expenditure.mp. (927)

72 health care expenditures.mp. (1766)

73 value of life.mp. (388)

74 societal benefit*.mp. (335)

75 economic evaluation.mp. or exp economic evaluation/ (211446)

76 economic analys*.mp. (5522)

77 economic assessment.mp. (775)

78 health economics.mp. or exp health economics/ (610100)

79 resource allocation.mp. or exp resource allocation/ (18574)

80 cost avoidance.mp. (404)

81 costs avoidance.mp. (9)

82 loss avoidance.mp. (37)

83 losses avoidance.mp. (1)

84 33 or 34 or 35 or 36 or 37 or 38 or 39 or 40 or 41 or 42 or 43 or 44 or 45 or 46or 47 or 48 or 49 or 50 or 51 or 52 or 53 or 54 or 55 or 56 or 57 or 58 or 59 or 60 or 61 or 62 or 63 or 64 or 65 or 66 or 67 or 68 or 69 or 70 or 71 or 72 or 73 or 74 or 75 or 76 or 77 or 78 or 79 or 80 or 81 or 82 or 83 (685916)

85 social-ecological.mp. (654)

86 1 or 2 or 3 or 4 or 5 or 6 or 7 or 8 or 9 or 10 or 11 or 12 or 13 or 14 or 15 or 16or 17 or 18 or 19 or 20 or 21 or 22 or 23 or 24 or 25 or 26 or 27 or 28 or 29 or 30 or 31 or 32 or 85 (1583112)

87 84 and 86 (25661)

***************************

**S7.** The four references included in this scoping review on the quantitative outcome of a One Health approach to address complex global health challenges, classified as studies illustrative of One Health thinking, but with multiple links between intervention and outcome.

| **Author and year** |  | **Reference** | **Geographical location** | **Study design** | **Health Issue addressed** | **One Health approach** | **Quantitative outcome** | **Methods used clearly stated** |
| --- | --- | --- | --- | --- | --- | --- | --- | --- |
| Friel; 2009 |  | Public health benefits of strategies to reduce greenhouse-gas emissions: food and agriculture | United Kingdom; Brazil | Mathematical model | Greenhouse gas production caused by food production and agriculture | Animal-environment | Non-monetary | Yes |
| McCubin; 2002 |  | Livestock ammonia management and particulate-related health benefits | United States of America | Other | Ammonia emissions and premature mortality | Animal-environment | Monetary and non-monetary | Partly |
| Shindell; 2012 |  | Simultaneously mitigating near-term climate change and improving human health and food security | Global | Economic analysis | Air pollution and premature mortality | Human-environment | Monetary and non-monetary | No |
| Shwiff; 2008 |  | Economic evaluation of an oral rabies vaccination program for control of a domestic dog-coyote rabies epizootic- 1995-2006 | United States of America | Economic analysis | Rabies | Human-animal | Monetary | Yes |

**S8.** The twelve references included in this scoping review on the quantitative outcome of a One Health approach to address complex global health challenges, and classified as mixed intervention studies. These references described disease control programs or outbreak investigations which included One Health interventions or thinking, but the quantitative outcome due to the One Health intervention could not be distinguished from the overall quantitative outcome.

| **Author and year** |  | **Reference** | **Geographical location** | **Study design** | **Health issue addressed** | **One Health approach** | **Quantitative outcome** | **Methods used clearly stated** |
| --- | --- | --- | --- | --- | --- | --- | --- | --- |
| **Evaluation of disease control programs** | | |  |  |  |  |  |  |
| Lapiz; 2012 |  | Implementation of an inter-sectoral program to eliminate human and canine rabies: the Bohol rabies prevention and elimination project | Philippines | Assessment of control program | Rabies | Human-animal | Non-monetary | Yes |
| Kamoltham; 2003 |  | Elimination of human rabies in a canine endemic province in Thailand: five-year programme | Thailand | Assessment of control program | Rabies | Human-animal | Non-monetary | Yes |
| Morel; 2005 |  | Cost effectiveness analysis of strategies to combat malaria in developing countries | Two sub-Saharan African regions | Economic analysis | Malaria | Human-environment | Monetary | Partly |
| Mwacalimba; 2013 |  | Cost benefit analysis of tuberculosis control in wildlife–livestock interface areas of Southern Zambia | Zambia | Economic analysis | Bovine Tuberculosis | Human-animal | Monetary | Yes |
| Ndeffo Mbah; 2013 |  | Cost-effectiveness of a community-based intervention for reducing the transmission of *Schistosoma haematobium* and HIV in Africa | Zimbabwe | Mathematical model | Schistosomiasis and consequent HIV infections | Human-environment | Monetary | Partly |
| Utzinger; 2002 |  | The economic payoffs of integrated malaria control in the Zambian copperbelt between 1930 and 1950 | Zambia | Economic analysis | Malaria | Human-environment | Monetary and non-monetary | Yes |
| Wang; 2009 |  | A strategy to control transmission of *Schistosoma japonicum* in China | China | Controlled trial | Schistosomiasis | Human-animal-environment | Non-monetary | Yes |
| Xiao-Nong; 2005 |  | An economic evaluation of the national schistosomiasis control programme in China from 1992 to 2000 | China | Economic analysis | Schistosomiasis | Human-animal-environment | Monetary and non-monetary | Yes |
| Yu; 2013 |  | Impact and cost-effectiveness of a comprehensive Schistosomiasis japonica control program in the Poyang Lake Region of China | China | Case-control | Schistosomiasis | Human-animal-environment | Monetary | Yes |
| **Outbreak investigation or assessment** | | | |  |  |  |  |  |
| Baly; 2012 |  | Costs of dengue prevention and incremental cost of dengue outbreak control in Guantanamo, Cuba | Cuba | Economic analysis | Dengue | Human-environment | Monetary | Yes |
| Basant Sharme; 2010 |  | Reemergence of rabies in Chhukha district, Bhutan, 2008 | Bhutan | Case report | Rabies | Human-animal | Monetary | Yes |
| van Asseldonk 2013 |  | Economic assessment of Q fever in the Netherlands | The Netherlands | Mathematical modelling | Q fever | Human-animal | Monetary and non-monetary | No |

**S9.** The six references included in this scoping review on the quantitative outcome of a One Health approach to address complex global health challenges, and classified as environmental studies since the main outcome was environmental (or a component thereof) health

| **Author and year** | **Reference** | **Geographical location** | **Study design** | **Health issue addressed** | **One Health approach** | **Quantitative outcome** | **Method used clearly explained** |
| --- | --- | --- | --- | --- | --- | --- | --- |
| Ferguson; 2013 | Sustainability of holistic and conventional cattle ranching in the seasonally dry tropics of Chiapas, Mexico | Mexico | Case-control | Environmental sustainability | Animal-environment | Non-monetary | Yes |
| Holzschuh; 2010 | How do landscape composition and configuration, organic farming and fallow strips affect the diversity of bees, wasps and their parasitoids? | Germany | Case-control | Diversity of bees, wasps and parasitoids | Animal-environment | Non-monetary | Yes |
| Hospido; 2005 | The environmental impact of mastitis: a case study of dairy herds | Spain | Life Cycle Assessment | Environmental health | Animal-environment | Non-monetary | Yes |
| Petersen; 2014 | Mussels as a tool for mitigation of nutrients | Denmark | Case report | Water quality | Animal-environment | Monetary | Yes |
| Sandom; 2013 | Rooting for Rewilding: Quantifying Wild Boar's *Sus scrofa* Rooting Rate in the Scottish Highlands | Scotland | Controlled trial | Woodland regeneration and reinvigoration | Animal-environment | Monetary | Yes |
| Scernewski; 2012 | Zebra Mussel Farming in the Sczecin (Oder) Lagoon: water-quality objectives and cost-effectiveness | Oder River and Lagoon | Economic analysis | Water quality | Animal-environment | Monetary | Yes |

**S10.** The fourteen references included in a scoping review on the quantitative outcome of a One Health approach to address complex global health challenges, and that described an abiotic disease agent

| **Author and year** | **Reference** | **Geographical location** | **Study design** | **Health issue addressed** | **One Health approach** | **Quantitative outcome** | **Methods used clearly explained** |
| --- | --- | --- | --- | --- | --- | --- | --- |
| Bauman; 2001 | The epidemiology of dog walking: an unmet need for human and canine health | Australia | Cross-sectional | Cardiac disease | Human-animal | Non-monetary | No |
| Dallat; 2013 | Urban greenways have the potential to increase physical activity levels cost-effectively | Ireland | Economic analysis | Cancer, diabetes and cardiac disease | Human-environment | Monetary and non-monetary | Yes |
| Howard; 2006 | Risk assessment of Arsenic mitigation options in Bangladesh | Bangladesh | Risk assessment | Arsenic poisoning and gastrointestinal disease | Human-environment | Non-monetary | Yes |
| Kushner; 2006 | The PPET study: people and pets exercising together | USA | Controlled trial | Obesity | Human-animal | Non-monetary | Yes |
| Li; 2004 | Quantifying the human health benefits of curbing air pollution in Shanghai | China | Economic analysis | Respiratory and cardiac disease and overall mortality | Human-environment | Monetary | Yes |
| Li; 2011 | Assessing the co-benefits of greenhouse gas reduction: health benefits of particulate matter related inspection and maintenance programs in Bangkok, Thailand | Thailand | Risk assessment and economic analysis | Respiratory and cardiac disease and overall mortality | Human-environment | Monetary and non-monetary | Yes |
| McCollum; 2013 | Climate policies can help resolve energy security and air pollution challenges | Not specified | Other | Human health | Human-environment | Non-monetary | No |
| Orozco; 2012 | Tackling challenges to farmers' health and agro-ecosystem sustainability in highland Ecuador | Ecuador | Cross-sectional | Pesticide poisoning and neurological disease | Human-environment | Non-monetary | Yes |
| Papa; 2013 | How green are environmental technologies? A new approach for a global evaluation: the case of WWTP effluents ozonation | Not specified | Risk assessment | Human health | Human-environment | Monetary | No |
| Shah; 2001 | Rains-Asia model application to China – policy implications for sulfur control | China | Economic analysis | Overall morbidity and mortality | Human-environment | Monetary | No |
| van Wezel; 2008 | Societal Cost–Benefit analysis for soil remediation in the Netherlands | The Netherlands | Economic analysis | Respiratory disease and intoxication | Human-environment | Monetary and non-monetary | Yes |
| Voorhees; 2000 | An Ex Post Cost-Benefit analysis of the Nitrogen Dioxide air pollution control program in Tokyo | Japan | Economic analysis | Respiratory disease | Human-environment | Monetary | Yes |
| Wilkinson; 2009 | Public health benefits of strategies to reduce greenhouse-gas emissions: household energy | United Kingdom | Risk assessment | Respiratory disease | Human-environment | Non-monetary | Yes |
| Wilkinson; 2009 | Public health benefits of strategies to reduce greenhouse-gas emissions: household energy | India | Risk assessment | Respiratory and cardiac disease | Human-environment | Non-monetary | Yes |

**S11.** The forty references included in this scoping review on the quantitative outcome of a One Health approach to address complex global health challenges, and that described a biotic disease agent not considered a neglected tropical disease

| **Author and year** |  | **Reference** | **Geographical location** | **Study design** | **Health issue addressed** | **One Health approach** | **Quantitative outcome** | **Methods used clearly explained** |
| --- | --- | --- | --- | --- | --- | --- | --- | --- |
| Aikins; 1998 |  | The Gambian national impregnated bednet programme: costs, consequences and net cost-effectiveness | Gambia | Economic analysis | Malaria | Human-environment | Monetary and non-monetary | Yes |
| Akhavan; 1999 |  | Cost-effective malaria control in Brazil cost-effectiveness of a Malaria control program in the Amazon Basin of Brazil, 1988-1996 | Brazil | Assessment of control program | Malaria | Human-environment | Monetary and non-monetary | Yes |
| Barber; 2010 |  | Economic cost analysis of West Nile virus outbreak, Sacramento County, California, USA, 2005 | USA | Assessment of control program | West Nile | Human-animal | Monetary | Yes |
| Bechir; 2004 |  | Approche Novatrice des vaccinations en Santé Publique et en Médecine Vétérinaire chez les pasteurs nomades au Tchad: expériences et coûts [An innovative approach combining human and animal vaccination campaigns in nomadic settings of Chad: experiences and costs] | Chad | Economic analysis | Multiple | Human-animal | Monetary | Yes |
| Coelho; 2011 |  | Cost-benefit analysis of sheep and goat brucellosis vaccination with Rev.1 in the North of Portugal from 2000 to 2005 | Portugal | Economic analysis | Brucellosis | Human-animal | Monetary | Yes |
| Cohen; 1999 |  | Cost-effectiveness of Bovine Spongiform Encephalopathy screening | United Kingdom, Switzerland & France | Economic analysis | Bovine Spongiform Encephalopathy and variant Creutzfeldt-Jakob Disease | Human-animal | Monetary and non-monetary | Yes |
| Gatton; 2010 |  | Interrupting malaria transmission: quantifying the impact of interventions in regions of low to moderate transmission | Australia | Mathematical model | Malaria | Human-environment | Non-monetary | Yes |
| Gellynck; 2008 |  | Economics of reducing Campylobacter at different levels within the Belgian poultry meat chain | Belgium | Economic analysis | Campylobacteriosis | Human-animal | Monetary and non-monetary | Yes |
| Given; 2006 |  | Regional public health cost estimates of contaminated coastal waters: a case study of gastroenteritis at Southern California beaches | USA | Case-series | Gastrointestinal disease | Human-environment | Monetary and non-monetary | No |
| Goldbach; 2005 |  | A cost–benefit analysis of Salmonella-control strategies in Danish pork production | Denmark | Economic analysis | Salmonellosis | Human-animal | Monetary | Yes |
| Goodman; 1999 |  | Cost-effectiveness of malaria control in sub-Saharan Africa | Hypothetical population from West Africa | Economic analysis | Malaria | Human-environment | Monetary | Yes |
| Goodman; 2001 |  | Comparison of the cost and cost-effectiveness of insecticide-treated bednets and residual house-spraying in KwaZulu-natal South Africa | South Africa | Challenge trial | Malaria | Human-environment | Monetary and non-monetary | Yes |
| Havelaar; 2007 |  | Effectiveness and efficiency of controlling Campylobacter on broiler chicken meat | The Netherlands | Mathematical model | Campylobacteriosis | Human-animal | Monetary and non-monetary | Yes |
| Hunter; 2012 |  | The effect of risk perception on public preferences and willingness to pay for reductions in the health risks posed by toxic cyanobacterial blooms | Scotland | Economic analysis | Cyanotoxin exposure | Human-environment | Monetary | Yes |
| Jensen; 2013 |  | Vaccination of poultry against Campylobacter in the EU – what are the benefits? | European Union | Economic analysis | Campylobacteriosis | Human-animal | Monetary | Yes |
| Johnson; 2008 |  | A framework for valuing the health benefits of improved bathing water quality in the river Irvine catchment | Scotland | Mathematical model and economic analysis | Gastrointestinal disease | Human-environment | Monetary | Yes |
| Kangas; 2007 |  | Costs of two alternative Salmonella control policies in Finnish broiler production | Finland | Economic analysis | Salmonellosis | Human-animal | Monetary | Yes |
| Korsgaard; 2009 |  | The effects, costs and benefits of Salmonella control in the Danish table-egg sector | Denmark | Economic analysis | Salmonellosis | Human-animal | Monetary and non-monetary | Yes |
| Lake; 2013 |  | Cost-effectiveness of interventions to control Campylobacter in the New Zealand poultry meat food supply | New Zealand | Economic analysis | Campylobacteriosis | Human-animal | Monetary | Yes |
| Mangen; 2007 |  | Cost-utility analysis to control Campylobacter on chicken meat – Dealing with data limitations | The Netherlands | Economic analysis | Campylobacteriosis | Human-animal | Monetary | Yes |
| Miller; 2005 |  | Influence of Salmonella in pigs preharvest and during pork processing on human health costs and risks from pork | USA | Other | Salmonellosis | Human-animal | Monetary and non-monetary | Yes |
| Morlando; 2012 |  | Reduction in Lyme disease risk as an economic benefit of habitat restoration | USA | Economic analysis | Lyme disease | Human-environment | Monetary | Yes |
| Mueller; 2008 |  | Cost effectiveness analysis of insecticide-treated net distribution as part of Togo Integrated Child Health Campaign | Togo | Economic analysis | Malaria | Human-environment | Monetary and non-monetary | Yes |
| Mulligan; 2008 |  | Costs and effects of the Tanzanian national voucher scheme for insecticide-treated nets | Tanzania | Economic analysis | Malaria | Human-environment | Monetary and non- monetary | Yes |
| Ngnikam; 2012 |  | Water, wastes, and children's health in low-income neighbourhoods of Yaoundé | Cameroon | Cohort | Gastrointestinal disease | Human-environment | Non-monetary | Yes |
| Persson;1992 |  | The economic impact of poultry-borne salmonellosis: how much should be spent on prophylaxis? | England, Wales and Sweden | Economic analysis | Salmonellosis | Human-animal | Monetary | No |
| Pulkki-Brännström; 2012 |  | Cost and cost effectiveness of long-lasting insecticide-treated bed nets – a model-based analysis | Not specified | Economic analysis | Malaria | Human-environment | Monetary and non-monetary | Yes |
| Riedel; 2010 |  | Geographical patterns and predictors of malaria risk in Zambia: Bayesian geostatistical modelling of the 2006 Zambia national malaria indicator survey (ZMIS) | Zambia | Cross-sectional | Malaria | Human-environment | Non-monetary | Partly |
| Romero-Barrios; 2013 |  | Quantitative microbiological risk assessment (QMRA) of food-borne zoonoses at the European level | European Union | Mathematical model | Campylobacteriosis | Human-animal | Non-monetary | Yes |
| Romero-Barrios; 2013 |  | Quantitative microbiological risk assessment (QMRA) of food-borne zoonoses at the European level | European Union | Mathematical model | Salmonellosis | Human-animal | Non-monetary | Yes |
| Roth; 2003 |  | Human health benefits from livestock vaccination for brucellosis – case study | Mongolia | Economic analysis | Brucellosis | Human-animal | Monetary and non-monetary | Yes |
| Sardar; 2013 |  | An optimal cost effectiveness study on Zimbabwe cholera seasonal data from 2008–2011 | Zimbabwe | Economic analysis | Cholera | Human-environment | Monetary and non-monetary | Yes |
| Schelling; 2007 |  | Human and animal vaccination delivery to remote nomadic families, Chad | Chad | Assessment of control program | Multiple | Human-animal | Monetary and non-monetary | Partly |
| Simon; 2012 |  | Intersectoral health action in Tanzania – determinants and policy implications | Tanzania | Mathematical model and economic analysis | Multiple | Human-environment | Non-monetary | Partly |
| Smithuis; 2013 |  | The effect of insecticide-treated bed nets on the incidence and prevalence of malaria in children in an area of unstable seasonal transmission in western Myanmar | Myanmar (Burma) | Randomized control trial and economic analysis | Malaria | Human-environment | Monetary and non-monetary | Yes |
| Wegener; 2003 |  | Salmonella control programs in Denmark | Denmark | Assessment of control program | Salmonellosis | Human-animal | Monetary and non-monetary | Yes |
| Withee; 2009 |  | Streamlined analysis for evaluating the use of pre-harvest interventions intended to prevent Escherichia coli O157:H7 illness in humans | USA | Economic analysis | Escherichia coli | Human-animal | Monetary | Yes |
| Wyss; 1996 |  | Central America regional screwworm eradication | Central America | Economic analysis | Screwworm | Animal-environment | Monetary | No |
| Yhdego; 1988 |  | Malaria control in Tanzania | Tanzania | Economic analysis | Malaria | Human-environment | Monetary | Yes |

**S12.** The 32 references included in this scoping review on the quantitative outcome of a One Health approach to address complex global health challenges, and that described a biotic disease agent considered a neglected tropical disease

| **Author and year** |  | **Reference** | **Geographical location** | **Study design** | **Health issue addressed** | **One Health approach** | **Quantitative outcome** | **Methods used clearly described** |
| --- | --- | --- | --- | --- | --- | --- | --- | --- |
| Attanasio’; 1984 |  | Cost-effectiveness analysis of *Echinococcus hydatidosis* eradication project in Sardinia | Italy | Economic analysis | Echinococcosis | Human-animal | Monetary and non-monetary | Yes |
| Aubert; 1999 |  | Costs and benefits of rabies control in wildlife in France | France | Assessment of control program and economic analysis | Rabies | Human-animal | Monetary and non-monetary | Partly |
| Bögel; 1990 |  | Economics of human and canine rabies elimination: guidelines for programme orientation | Developing countries | Mathematical model | Rabies | Human-animal | Monetary | Yes |
| Budke; 2005 |  | Economic effects of echinococcosis in a disease-endemic region of the Tibetan plateau | China | Economic analysis | Echinococcosis | Human-animal | Monetary | Yes |
| de Barros Ferreira Pinto; 2011 |  | Avaliacao do custo-beneficio das atividades de prevencao da raiva humana e das atividades de controle da raiva caninca no municipio de mogi guacu, estado de Sao Paulo, no periodo de 2000 a 2004 [Cost-benefit analysis of the activities for human rabies prevention and the activities for canine rabies control in Mogi Guaco Municipality, State of Sao Paulo from 2000-2004] | Brazil | Economic analysis | Rabies | Human-animal | Monetary | Yes |
| Diaz; 2012 |  | Preventing dengue at the local level in Havana City | Cuba | Other | Dengue | Human-environment | Non-monetary | No |
| Fishbein; 1991 |  | Rabies control in the Republic of the Philippines: benefits and costs of elimination | Philippines | Economic analysis | Rabies | Human-animal | Monetary | Yes |
| Fitzpatrick; 2014 |  | Cost-effectiveness of canine vaccination to prevent human rabies in rural Tanzania | Tanzania | Mathematical model | Rabies | Human-animal-environment | Monetary and non-monetary | Yes |
| Gilioli; 2007 |  | Adaptive eco-social system sustainability enhancement in sub-Saharan Africa | Italy | Other | Malaria, trypanosomiasis and tick-borne diseases | Human-animal-environment | Non-monetary | No |
| Gray; 2009 |  | A cluster-randomised intervention trial against *Schistosoma japonicum* in the People's Republic of China: bovine and human Transmission | China | Randomized control trial | Schistosomiasis | Human-animal | Non-monetary | Yes |
| Häsler; 2014 |  | A One Health framework for the evaluation of rabies control programmes – a case study from Colombo city Sri Lanka | Sri Lanka | Economic analysis | Rabies | Human-animal | Monetary and non-monetary | Yes |
| Hegglin; 2013 |  | Control of *Echinococcus multilocularis*: strategies, feasibility and cost-benefit analyses | Not specified (Western Europe) | Mathematical model | Echinococcosis | Human-animal | Monetary | No |
| Jiménez; 2002 |  | Progress in control of cystic echinococcosis in La Rioja, Spain: decline in infection prevalences in human and animal hosts and economic costs and benefits | Spain | Assessment of control program | Echinococcosis | Human-animal | Monetary and non-monetary | Yes |
| Kajunguri; 2014 |  | Modelling the use of insecticide-treated cattle to control Testse and in a multi-host population *Trypanosoma brucei rhodesiense* | Sub-Saharan Africa | Mathematical model | Trypanosomiasis | Human-animal | Non-monetary | Yes |
| Mazloumi Gavgani; 2002 |  | Effect of insecticide-impregnated dog collars on incidence of zoonotic visceral leishmaniasis in Iranian children: a matched-cluster randomised trial | Iran | Randomized control trial | Visceral leishmaniasis | Human-animal | Non-monetary | Yes |
| McConnell; 2003 |  | Guidelines on the cost-effectiveness of larval control programs to reduce dengue transmission in Puerto Rico | Puerto Rico | Economic analysis | Dengue | Human-environment | Monetary | Yes |
| Ministère de la Solidarité, de la Santé et de la Protection Sociale Française (Direction générale de la Santé) ; 1989 |  | Étude coût-bénéfice de la prophylaxie médicale de la rage vulpine [Cost-benefit analysis of oral rabies vaccination in foxes] | France | Economic analysis | Rabies | Human-animal | Monetary | Partly |
| Ocampoa; 2014 |  | Reduction in dengue cases observed during mass control of *Aedes* (Stegomyia) in street catch basins in an endemic urban area in Colombia | Colombia | Controlled trial | Dengue | Human-environment | Non-monetary | Partly |
| Orellano; 2008 |  | Cost-benefit analysis of vector control in areas of potential dengue transmission | Argentina | Economic analysis | Dengue | Human-environment | Monetary | Yes |
| Orellano; 2013 |  | Cost-effectiveness of prevention strategies for American tegumentary leishmaniasis in Argentina | Argentina | Economic analysis | Tegumentary leishmaniasis | Human-environment | Monetary and non-monetary | Yes |
| Rojas de Arias; 1999 |  | Chagas disease vector control through different intervention modalities in endemic localities of Paraguay | Paraguay | Controlled trial | Chagas disease | Human-environment | Non-monetary | Yes |
| Schofield; 1991 |  | A cost-benefit analysis of Chagas disease control | Countries in South America | Economic analysis | Chagas disease | Human-environment | Monetary and non-monetary | Yes |
| Shwiff; 2011 |  | A retrospective economic analysis of the Ontario red fox oral rabies vaccination programme | Canada | Economic analysis | Rabies | Human-animal | Monetary | Yes |
| Shwiff; 2013 |  | Bioeconomic modelling of raccoon rabies spread management impacts in Quebec, Canada | Canada | Mathematical model | Rabies | Human-animal | Monetary | Yes |
| Suaya; 2007 |  | Cost-effectiveness of annual targeted larviciding campaigns in Cambodia against the dengue vector *Aedes aegypti* | Cambodia | Economic analysis | Dengue | Human-environment | Monetary and non-monetary | Yes |
| Tenzin; 2012 |  | Human and animal rabies prevention and control cost in Bhutan, 2001-2008: the cost-benefit of dog rabies elimination | Bhutan | Economic analysis | Rabies | Human-animal | Monetary | Yes |
| Townsend; 2013 |  | Designing programs for eliminating canine rabies from islands: Bali, Indonesia as a case study | Indonesia | Mathematical model | Rabies | Human-animal | Monetary and non-monetary | Yes |
| Tsunoda; 2013 |  | Field trial on a novel control method for the dengue vector, *Aedes aegypti* by the systematic use of Olyset Net and pyriproxyfen in Southern Vietnam | Vietnam | Case-control | Dengue | Human-environment | Non-monetary | Yes |
| Uhaa; 1992 |  | Benefits and costs of using an orally absorbed vaccine to control rabies in raccoons | United States of America | Economic analysis | Rabies | Human-animal | Monetary | Yes |
| Vazquez-Prokopec; 2009 |  | Cost-effectiveness of Chagas disease vector control strategies in northwestern Argentina | Argentina | Economic analysis | Chagas disease | Human-environment | Monetary and non-monetary | Yes |
| Wilson; 2005 |  | Cost-effectiveness of Chagas disease interventions in Latin America and the Caribbean: Markov models | Countries in Latin America and the Caribbean | Mathematical model | Chagas disease | Human-environment | Monetary and non-monetary | Yes |
| Zinsstag; 2009 |  | Transmission dynamics and economics of rabies control in dogs and humans in an African city | Chad | Mathematical model | Rabies | Human-animal | Monetary | Yes |

**S13: Sources (with respective links) and definitions that were used for the extraction of additional parameters relevant to the review**

| **Variable** | **Source** | **Link** |
| --- | --- | --- |
| Continent | World Trade Organization | <https://www.wto.org/english/res_e/statis_e/its2014_e/its2014_e.pdf>) |
| Country development status | United Nations | <http://www.un.org/en/development/desa/policy/wesp/wesp_current/2014wesp_country_classification.pdf> |
| Country income status | World Bank | <http://data.worldbank.org/about/country-and-lending-groups> |
| Disease agent | World Health Organization | <http://www.who.int/mediacentre/factsheets/en/> |
|  | International Statistical Classification of Diseases and Related Health Problems 10th Revision | <http://apps.who.int/classifications/icd10/browse/2016/en> |
| Neglected tropical disease | World Health Organization | <http://www.who.int/neglected_diseases/diseases/summary/en/> |
| Transmission type | Ontobee Pathogen Transmission Ontology | <http://www.ontobee.org/ontology/catalog/TRANS?iri=http://www.w3.org/2002/07/owl%23Class> |
